# Diagnostic Yield of Endometrial Sampling by Age in Premenopausal Women with Abnormal Uterine Bleeding: A Retrospective Cross-Sectional Study

**DOI:** 10.3390/jcm15218523

**Published:** 2026-11-02

**Authors:** Izabella Erdelean, Dragoș Erdelean, Răzvan Roșca, Andrei Pescaru, Cosmin Cîtu, Denis Mihai Șerban, Victor Bogdan Buciu, Anca-Raluca Dinu, Nilima Rajpal Kundnani, Sebastian Ciurescu

**Affiliations:** 1 Department of Obstetrics and Gynecology, “Victor Babeș” University of Medicine and Pharmacy, Eftimie Murgu Square, No. 2, 300041 Timișoara, Romania; 2 Department III Functional Sciences, Discipline of Medical Informatics and Biostatistics, “Victor Babeș” University of Medicine and Pharmacy, 300041 Timișoara, Romania; 3 Doctoral School, “Victor Babeș” University of Medicine and Pharmacy, Eftimie Murgu Square, No. 2, 300041 Timișoara, Romania; 4 Obstetrics-Gynecology Clinic IV, Timișoara Municipal Emergency Clinical Hospital, 300202 Timișoara, Romania; 5 Department XVI, Medical Recovery, “Victor Babeș” University of Medicine and Pharmacy, 300041 Timișoara, Romania; 6 Research Center for Assessment of Human Motion and Functionality and Disability, “Victor Babeș” University of Medicine and Pharmacy, Eftimie Murgu Square, No. 2, 300041 Timișoara, Romania; 7 “Pius Brinzeu” Emergency Clinical County Hospital, Bld Liviu Rebreanu, No. 156, 300723 Timișoara, Romania; 8 Department VI—Cardiology, “Victor Babeș” University of Medicine and Pharmacy, 300041 Timișoara, Romania; 9 Research Centre of Timișoara Institute of Cardiovascular Diseases, “Victor Babeș” University of Medicine and Pharmacy, 300041 Timișoara, Romania

**Keywords:** abnormal uterine bleeding, endometrial biopsy, endometrial hyperplasia, age threshold, cross-sectional study, diagnostic yield

## Abstract

**Background**: An age of 45 years is often used to guide endometrial assessment in abnormal uterine bleeding (AUB), but age is only one component of clinical risk. We described the relationship between age and histopathological yield among premenopausal women who had undergone endometrial sampling. **Methods**: This single-centre retrospective cross-sectional study analysed the index sampling episodes of 824 women with AUB documented as premenopausal or perimenopausal (January 2022–May 2026). The clinically more consequential endpoint was endometrial intraepithelial neoplasia (EIN)/atypical hyperplasia or carcinoma; the primary statistical outcome was a broader composite that also included hyperplasia without atypia. Modified Poisson regression estimated the adjusted association between age ≥45 years and the primary outcome. Discrimination of age alone and of an exploratory model using variables available before biopsy was compared in 776 complete cases with bootstrap internal validation. **Results**: EIN/carcinoma occurred in 4/361 (1.1%) women aged <45 years and 25/463 (5.4%) aged ≥45 years (crude RR 4.87, 95% CI 1.71–13.88). The broad outcome occurred in 24/361 (6.6%) and 57/463 (12.3%), respectively (absolute difference 5.7 percentage points, 95% CI 1.6–9.6; adjusted RR 1.62, 95% CI 1.01–2.61; *p* = 0.047). Results were similar across sensitivity analyses (adjusted RRs 1.52–1.68). With age coded as ≥45 years in both models, the optimism-corrected AUC was 0.686 for the exploratory pre-biopsy model and 0.575 for age alone (difference 0.111, 95% CI 0.060–0.196). **Conclusions**: Among premenopausal women selected for endometrial sampling, histopathological yield increased with age, and most EIN/carcinoma cases occurred at age ≥45 years, but the adjusted association for the broad outcome was imprecise, and age alone had limited discrimination. Model performance was assessed only internally and only in sampled women; this study cannot establish a biopsy rule for all women with AUB because women not sampled were not observed.

## 1. Introduction

Abnormal uterine bleeding (AUB) is an umbrella symptom that includes abnormalities of menstrual frequency, regularity, duration and volume as well as intermenstrual bleeding. The International Federation of Gynaecology and Obstetrics (FIGO) recommends a two-stage approach: System 1 standardises the description of bleeding symptoms, whereas System 2 classifies potential causes using PALM-COEIN (polyp, adenomyosis, leiomyoma, malignancy and hyperplasia; coagulopathy, ovulatory dysfunction, endometrial, iatrogenic and not otherwise classified) [1,2]. This terminology is important because AUB is not a single disease, and more than one structural or non-structural mechanism may coexist in the same patient [1,2].

Heavy or unpredictable bleeding impairs physical functioning, work productivity and emotional well-being [3,4], and barriers to seeking care, including normalisation of symptoms, embarrassment and variable access to specialist services, mean that the women who reach endometrial sampling represent a clinically selected subset rather than the full community burden of AUB [4,5].

The principal oncological purpose of endometrial assessment is the timely detection of endometrial hyperplasia, endometrial intraepithelial neoplasia (EIN)/atypical hyperplasia and carcinoma. Endometrial carcinogenesis is heterogeneous, but prolonged oestrogenic stimulation without adequate progestogenic opposition is a central pathway for many endometrioid lesions [6]. Obesity contributes through peripheral aromatisation, insulin resistance, inflammation and altered adipokine signalling; diabetes, polycystic ovary syndrome (PCOS), chronic anovulation and nulliparity may further identify women with prolonged or cumulative hormonal and metabolic risk [6,7,8]. These factors are interrelated, which makes single-variable decision rules clinically attractive but biologically incomplete [6,7,8].

Clinical guidelines therefore combine age with risk context. The American College of Obstetricians and Gynaecologists (ACOG) recommends endometrial tissue sampling as a first-line test in patients with AUB older than 45 years and recommends sampling in younger patients when there is unopposed oestrogen exposure, persistent AUB or failed medical management [9,10]. The National Institute for Health and Care Excellence (NICE) does not treat age alone as a universal indication in heavy menstrual bleeding; it advises considering biopsy, generally at hysteroscopy, for women at higher risk, including those with persistent intermenstrual or irregular bleeding, infrequent heavy bleeding with obesity or PCOS, tamoxifen exposure, or unsuccessful treatment [11]. Thus, major guidelines share the goal of detecting important pathology but operationalise the balance between age, symptoms and risk factors differently [9,10,11].

That balance is difficult because serious pathology is uncommon in most premenopausal AUB populations. A systematic review of 65 studies including 29,059 premenopausal women estimated the risk of endometrial cancer at 0.33% and the combined risk of cancer or atypical hyperplasia at 1.31%; risk was lower with heavy menstrual bleeding alone than with intermenstrual bleeding [12]. However, estimates vary with age distribution, referral pathway, inclusion criteria, outcome definition and whether all symptomatic women or only women already selected for biopsy are studied [12,13,14,15]. In a more selected cohort of women aged 45 years or younger who underwent sampling, atypical hyperplasia or cancer occurred in 6.0%, and nulliparity, PCOS and multiple polyps were associated with the outcome [13]. Other prediction studies have similarly implicated age, obesity or diabetes, reproductive history, bleeding pattern and ultrasonographic features while showing that no single characteristic reliably separates all women with and without disease [14,15].

Endometrial sampling itself is not a uniform reference procedure. Office aspiration biopsy, dilatation and curettage, and hysteroscopically directed biopsy differ in invasiveness, anaesthetic requirements, tissue volume and ability to target focal lesions [11,16,17,18]. Meta-analyses support high diagnostic accuracy for endometrial cancer when adequate tissue is obtained, but inadequate or failed samples are clinically relevant, and blind techniques may be less sensitive for focal intracavitary pathology [16,17,18]. Transvaginal ultrasonographic endometrial thickness can contribute to assessment, yet interpretation before menopause is complicated by cycle-related variation, irregular ovulation and the absence of a universally accepted diagnostic cutoff comparable with that used in postmenopausal bleeding [11,14,19].

An age of 45 years is therefore best understood as a pragmatic clinical decision threshold rather than a demonstrated biological discontinuity. Chronological age is correlated with cumulative exposure and with the menopausal transition, but it is not equivalent to menopausal status; women of the same age may differ substantially in ovulatory function, metabolic risk, bleeding phenotype and endometrial appearance [1,6,9,10,11,12,13,14,15]. Despite the wide use of this threshold, few contemporary data describe how the yield of hyperplasia, EIN and carcinoma changes across narrow age strata in premenopausal women, particularly from Central and Eastern European practice, and many cohorts report a single pooled outcome without separating the clinically more consequential EIN/carcinoma endpoint [12,13,14,15]. Because associations of age and of metabolic and reproductive factors with endometrial pathology are already established [6,7,8,12,13,14,15], the present study does not propose or validate a new threshold or biopsy-selection strategy. Its contribution is descriptive: it provides an explicitly defined denominator of consecutively sampled women from a Romanian tertiary service, reports the broad and the EIN/carcinoma endpoints across age strata and as a continuous function of age, and keeps the description of the age–yield relationship separate from the assessment of predictive discrimination.

The primary objective was descriptive: to estimate the age–yield relationship among premenopausal women already sampled for AUB, specifically by comparing the observed yield of significant endometrial pathology at ages <45 and ≥45 years. Secondary descriptive objectives were to report narrower age strata and the EIN/carcinoma endpoint, examine age continuously and estimate adjusted associations. A distinct, exploratory predictive objective was to assess the internally validated discrimination and calibration of age alone versus a model using information available before biopsy. Because only sampled women were observed, none of these analyses evaluates the performance of an age-based biopsy policy.

## 2. Materials and Methods

### 2.1. Study Design and Reporting

This was a single-centre retrospective cross-sectional diagnostic-yield study of index endometrial sampling episodes; the outcome was the histopathological finding at the index sampling, and no longitudinal follow-up was analysed. Accordingly, the risk ratios (RRs) reported here are ratios of the proportions of women in whom pathology was detected at index sampling (prevalence ratios within the sampled population), not ratios of incident disease risk. All consecutive eligible procedures during the study period were considered. Reporting follows STROBE for observational studies and the RECORD extension for routinely collected health data [20,21]. Reporting matrices are provided in Supplementary Tables S1 and S2. The variables, coding rules, age categories and covariates of the adjusted model were defined in a structured data dictionary finalised before data abstraction (Section 2.6); no formal statistical analysis plan was registered or time-stamped. The comparison of <45 versus ≥45 years was designated as the main analysis; all other analyses are secondary or exploratory, and analyses added during peer review are identified as post hoc (Section 2.9).

### 2.2. Setting and Data Source

**Figure 1:**
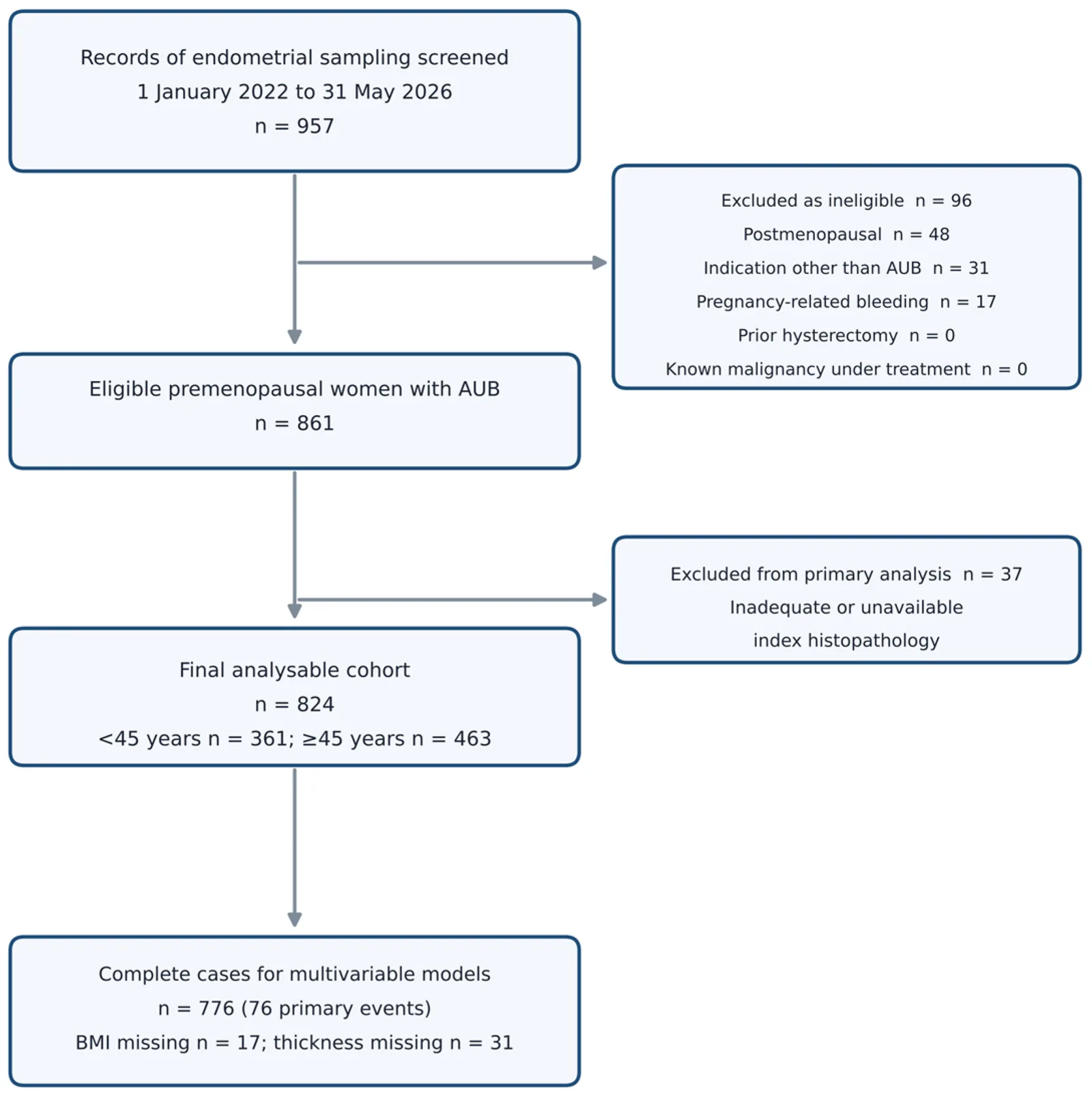
Participant screening and exclusions for the retrospective cross-sectional study.

The setting was the tertiary gynaecology service of Timișoara Municipal Emergency Clinical Hospital, Timișoara, Romania, which serves approximately 500,000 inhabitants. The study period was 1 January 2022 through 31 May 2026. Potentially eligible records were identified from operating-theatre, outpatient-procedure and pathology information systems; records of endometrial sampling procedures (Pipelle biopsy, dilatation and curettage or hysteroscopically directed biopsy) performed during the study period were consolidated at patient level before screening so that each of the 957 screened records represented one woman and, for women sampled more than once, only her first eligible procedure (Figure 1), and no other preliminary filter was applied. Clinical characteristics were extracted from electronic and paper medical records and linked to the final histopathology report using the hospital encounter identifier. The contemporary post-2021 period was selected to reduce heterogeneity related to the pandemic-era changes in referral, investigation and procedural access described in professional guidance for AUB services [22]. This choice was intended to limit, not to exclude, temporal heterogeneity; individual sampling dates were not available in the analytic dataset, so the stability of case mix and practice across the study years could not be examined.

The decision to sample and the choice of technique were made by the attending gynaecologist within guideline-influenced practice [9,10,11], and the specific indication for each procedure was not recorded as a structured variable. To examine whether younger women were sampled mainly because of additional risk factors, the frequency of selected guideline-related characteristics other than age (persistent AUB, failed medical treatment, intermenstrual or mixed bleeding, risk factors for unopposed oestrogen exposure and a suspected polyp) was compared between age groups (Supplementary Table S3).

### 2.3. Participants

Eligible participants were consecutive women aged 18 years or older who were documented as premenopausal; were evaluated for AUB; and underwent Pipelle biopsy, dilatation and curettage, or hysteroscopically directed endometrial sampling during the study period. Premenopausal status required ongoing menstruation or clinician-documented premenopausal/perimenopausal status without 12 consecutive months of amenorrhoea unrelated to another cause. Menopausal status was taken from the clinical documentation at the sampling encounter. The analytic dataset did not record the information on which this classification was based (for example, the date of the last menstrual period, the current cycle pattern or hormonal measurements), so status could not be verified independently, and women with ongoing cycles could not be distinguished from perimenopausal women with irregular cycles or amenorrhoea. Because these categories may represent different hormonal and ovulatory states, a sensitivity analysis excluded women aged ≥50 years (Section 2.9).

Exclusion criteria were documented postmenopausal status; pregnancy-related bleeding or tissue; sampling performed exclusively for an indication other than AUB; prior hysterectomy; known endometrial malignancy under treatment; and unavailable or inadequate histopathology for the primary analysis. When more than one sampling episode occurred, only the first eligible episode was retained to preserve patient-level independence.

### 2.4. Exposure and Outcomes

The primary exposure was age at sampling, dichotomised as <45 versus ≥45 years to evaluate a commonly used clinical threshold [9,10]. ACOG refers to patients older than 45 years; ≥45 years was used as the operational definition. Only nine women were aged exactly 45.0 years, and an analysis using >45 versus ≤45 years is reported in Section 3.6. Predefined secondary categories were younger than 40, 40–44, 45–49 and 50 years or older. Exact age was retained for continuous analyses.

The primary outcome was a broad composite of hyperplasia without atypia, EIN/atypical hyperplasia or endometrial carcinoma. It was chosen because hyperplasia without atypia is also clinically actionable, usually prompting progestogen treatment and follow-up sampling, and because too few EIN/carcinoma events were expected for adjusted analysis. The more clinically consequential secondary endpoint comprised EIN/atypical hyperplasia or carcinoma. Because hyperplasia without atypia accounts for most broad-outcome events and differs in natural history and inter-observer reproducibility, the EIN/carcinoma endpoint is given equal prominence in the presentation and interpretation of the results. Proliferative, secretory, disordered proliferative, atrophic/inactive and other non-neoplastic findings, including benign polyps, were classified as benign. Classification was based on the final index histopathology report (Supplementary Table S4); no central blinded pathology rereview was performed.

Each final report was coded into one of the nine categories listed in Supplementary Table S4. Hyperplasia was coded as either without atypia or with atypia/EIN, consistent with the two-tier World Health Organization (WHO) classification [23], as reported consistently by the institutional pathology service throughout the study period; diagnoses were not reclassified retrospectively by the investigators. Disordered proliferative endometrium was coded as benign. The classification edition cited in individual reports, the wording of ambiguous reports and the presence of more than one finding in a specimen were not recorded in the analytic dataset, so these could not be analysed separately; each specimen was assigned a single final category, which, when more than one finding was reported, was the most severe lesion (carcinoma > EIN/atypical hyperplasia > hyperplasia without atypia > benign) (Supplementary Table S4).

### 2.5. Covariates

Clinical variables collected a priori included body mass index (BMI) and obesity (BMI ≥ 30 kg/m^2^), parity and nulliparity, diabetes mellitus, hypertension, polycystic ovary syndrome (PCOS), chronic anovulation, tamoxifen exposure, current smoking, AUB pattern, persistence of symptoms, failed medical treatment, haemoglobin concentration, endometrial thickness on ultrasonography, uterine fibroids, suspected endometrial polyps and sampling method. Selection of these variables for data collection was informed by endometrial cancer biology, guideline indications and published risk-factor studies [6,7,8,9,10,11,12,13,14,15]. Intermenstrual or mixed bleeding was coded as the primary high-risk bleeding pattern [11,12]. Irregular bleeding was not included in this category because the pooled evidence shows the clearest excess risk with intermenstrual bleeding [12]; because irregular bleeding is also recognised in guidance [11], a sensitivity analysis classified it as high risk as well.

Operational definitions, as specified in the study data dictionary, were as follows. The AUB pattern was the dominant bleeding phenotype in four categories (heavy menstrual, irregular, intermenstrual or mixed bleeding). Persistent AUB denoted AUB persistent or recurrent for at least 6 months. Failed medical treatment denoted AUB documented as persisting despite medical therapy; because the agents, duration, adherence and response criteria were not recorded, this variable represents documented persistent bleeding after treatment rather than confirmed treatment failure. Chronic anovulation denoted a documented chronic anovulatory pattern, including PCOS; all 98 women with PCOS were also coded as chronically anovulatory. Diabetes mellitus denoted documented pre-existing or type 2 diabetes recorded in the medical history; the diabetes type was not recorded as a separate variable. Tamoxifen exposure denoted current or previous use. BMI was the value recorded nearest to sampling. Obesity was defined as BMI ≥ 30 kg/m^2^, derived from the recorded BMI and used consistently in the descriptive and regression analyses; the clinically recorded obesity indicator, which classified five women with a BMI of exactly 30.0 kg/m^2^ as non-obese, was used only in a sensitivity analysis (Section 3.6). Endometrial thickness was the maximum double-layer thickness on transvaginal ultrasonography, measured at intake as part of the first evaluation at the referral centre and therefore before the decision to sample, haemoglobin the value closest before sampling, and a suspected polyp a polyp suspected before sampling.

The dataset did not contain the cycle day of ultrasonography, the handling of focal lesions or intracavitary fluid in thickness measurement, current or recent hormonal treatment at the time of sampling (progestogens, hormonal contraception or a levonorgestrel-releasing intrauterine system), infertility history, hereditary predisposition to endometrial cancer, or the date of sampling for individual women; these factors could therefore not be analysed, and the implications are considered in Section 4.7.

The covariates included in the adjusted association model were defined before analysis and comprised age ≥45 years, obesity, diabetes mellitus, chronic anovulation, nulliparity, high-risk bleeding pattern, endometrial thickness and sampling method. PCOS was not entered separately because it is a principal cause of chronic anovulation, and all 98 women with PCOS were also coded as chronically anovulatory; chronic anovulation was therefore used as the broader hormonal-exposure construct, avoiding two collinear terms.

### 2.6. Data Extraction and Quality Assurance

A structured data dictionary was finalised before abstraction. Two investigators independently extracted the data and verified eligibility, and discrepancies were resolved by consensus after review of the source record; agreement between extractors before resolution was not formally quantified. Derived variables and outcome groupings were generated using reproducible syntax. The hospital encounter identifier was used only to link clinical records with pathology reports; in the analytical dataset each woman was identified by a study code (AUB-0001 to AUB-0824). Direct identifiers were not exported to the analytical dataset, and the data-extraction process was consistent with Regulation (EU) 2016/679 and Romanian Law No. 190/2018 [24,25].

### 2.7. Bias

Consecutive inclusion and explicit eligibility rules were used to limit investigator-driven selection and misclassification, and source-record verification and explicit missing-data denominators address some information bias. The consequences of clinical selection for sampling, confounding by indication and differences in sampling method are considered in Section 4.7.

### 2.8. Study Size

No sampling fraction or post hoc power calculation was used. The study size was determined by including all consecutive eligible patients during the 53-month period. A total of 957 records were screened and 824 women with adequate index histopathology were included, yielding 81 primary events overall (76 among the complete cases used for multivariable adjustment; Section 2.9). With nine parameters, the adjusted model had approximately 8.4 events per parameter, below commonly used rules of thumb [26]; it was therefore interpreted descriptively and was not used to derive a clinical biopsy rule.

### 2.9. Statistical Analysis

Continuous variables are presented as mean (standard deviation) and categorical variables as number (percentage), with the number of women with available data shown for variables with missing values. Baseline characteristics are descriptive. Diagnostic yield was calculated with Wilson 95% confidence intervals and differences in yield with Newcombe hybrid score intervals [27]. Crude RRs were estimated from observed proportions. The primary adjusted association used modified Poisson regression with HC0 robust standard errors [28]; logistic regression gave a complementary adjusted OR. Both models used the same 776 complete cases, and the crude RR was re-estimated in these 776 women so that crude and adjusted estimates refer to the same population. *p* values are reported to three decimal places and confidence limits to two, and results are interpreted by the magnitude and precision of the estimates rather than by a significance threshold.

The adjusted association model contained age ≥45 years, obesity, diabetes, chronic anovulation, nulliparity, intermenstrual/mixed bleeding, endometrial thickness and two indicator terms for sampling method (reference: dilatation and curettage). The target was the association between age ≥45 years and detected pathology within the sampled population, conditional on metabolic and reproductive characteristics that may differ by age and are associated with endometrial pathology (obesity, diabetes, chronic anovulation and nulliparity). Bleeding pattern, endometrial thickness and sampling method were included as characteristics of the diagnostic pathway that may differ by age; because they may also be consequences of the pathology or markers of clinical suspicion, adjustment for them may absorb part of the age association. The assumed causal structure is shown in Supplementary Figure S1, and models omitting these variables are reported as sensitivity analyses. Covariate coefficients are presented for completeness and should not be interpreted as total effects of those factors [29]. Because selection for sampling and several covariates may be affected by age or by the pathology itself (Supplementary Figure S1), the adjusted RR is a conditional association within the sampled population and not an estimate of the causal effect of age; the crude RR and the models omitting pathway variables (Section 3.6) are therefore reported alongside it. Other collected variables were retained for descriptive analysis rather than chosen through univariable screening. Tamoxifen exposure occurred in only three women and was too sparse for stable adjustment. Haemoglobin was not modelled because it reflects the severity of bleeding rather than a determinant of endometrial pathology.

Continuous age was examined with a restricted cubic spline using four knots at the 5th, 35th, 65th and 95th age percentiles [30]; a likelihood-ratio test assessed departure from linearity. The spline was fitted in a logistic model because a logistic model yields predicted probabilities and confidence bands that remain between 0 and 1, whereas the modified Poisson model was used to estimate the adjusted RR; the two analyses therefore address different questions (the shape of the age–yield relationship versus a summary ratio at the 45-year threshold). The exploratory pre-biopsy model was a logistic regression model that contained age ≥45 years, obesity, diabetes, chronic anovulation, nulliparity, intermenstrual/mixed bleeding and endometrial thickness (linear, per 1 mm; a three-knot restricted cubic spline did not improve fit, likelihood-ratio *p* = 0.87) but excluded sampling method, which is decided within the diagnostic pathway; its full specification, including the intercept and coefficients, is given in Supplementary Table S5. In the receiver operating characteristic (ROC) analyses, age alone was entered as a continuous variable (years); this explains why its AUC differs from the AUC implied by the sensitivity and specificity of the binary 45-year cutoff, which equals (sensitivity + specificity)/2. The principal discrimination comparison used the same binary ≥45-year age term in the age-only and the pre-biopsy model because the clinical question is whether pre-biopsy information adds to the conventional 45-year rule; a comparison with continuous age in both models was secondary. AUC confidence intervals were obtained with the DeLong method [31].

Body mass index (BMI) was unavailable for 17 of 824 women (2.1%) and endometrial thickness for 31 (3.8%); no woman was missing both measurements. The adjusted association and exploratory pre-biopsy models were analysed in the same 776 complete cases (94.2% of the cohort), among whom 76 had the primary outcome. Complete and incomplete cases were compared by age group, sampling method and outcome (Supplementary Table S6). As a sensitivity analysis, missing BMI and endometrial thickness values were imputed by multiple imputation by chained equations with predictive mean matching (20 imputed datasets, 20 iterations each and five donors per missing value, with all model variables and the outcome in the imputation model and obesity derived from imputed BMI), and estimates were combined with Rubin’s rules [32]. Trace plots of the means and standard deviations of imputed values showed no trends across iterations, and imputed BMI and thickness values had distributions similar to the observed values. The analysis assumes that data were missing at random given the variables in the imputation model, which cannot be verified; the similarity of complete and incomplete cases (Supplementary Table S6) does not exclude missing-data bias. All tests were two-sided with α = 0.05.

Post hoc sensitivity analyses, added in response to peer review, examined the robustness of the age estimate by (i) using HC3 robust standard errors [33] and a nonparametric bootstrap (2000 resamples) for its confidence interval; (ii) omitting endometrial thickness, sampling method or both; (iii) replacing binary obesity with continuous BMI and modelling endometrial thickness with a three-knot restricted cubic spline; (iv) using the recorded obesity indicator instead of BMI-derived obesity; (v) multiple imputation (above); (vi) excluding women aged ≥50 years; (vii) defining the exposure as age >45 versus ≤45 years; and (viii) also classifying irregular bleeding as high risk. Multicollinearity was assessed with variance inflation factors and influential observations with dfbeta values, and sparse covariate combinations were inspected by cross-tabulation. Analyses restricted to women with documented ongoing menstrual cycles or adjusted for calendar year, which were suggested during review, could not be performed because these variables were not available in the analytic dataset.

Discrimination and calibration of the age-only and pre-biopsy models were internally validated by bootstrap resampling [34]. In each of 1500 patient-level bootstrap samples drawn from the 776 complete cases, both models were refitted, and their AUCs were calculated in the bootstrap sample and in the original data; the mean difference estimated the optimism, which was subtracted from the apparent AUC. The optimism-corrected AUC difference between the models was obtained in the same way; its 95% confidence interval was obtained with the location-shifted bootstrap method [35], in which the 2.5th and 97.5th percentiles of the apparent differences between the models refitted in each of the 1500 resamples are shifted by the estimated mean optimism; this method showed adequate coverage for Harrell’s bias-corrected estimates in simulation studies [35]. Calibration of the pre-biopsy model was assessed with a bootstrap-corrected calibration plot and with the optimism-corrected calibration-in-the-large (intercept) and calibration slope [36]. The corrected intercept and slope equal the mean values obtained when each bootstrap-refitted model was applied to the original data, and the 2.5th–97.5th percentiles of these values are reported as approximate uncertainty intervals. Decision-curve analysis [37] was not performed because net benefit estimated among women already selected for sampling would not represent the consequences of using the model to decide whether to sample.

The comparison of the broad outcome at <45 versus ≥45 years was the main analysis. The EIN/carcinoma endpoint, narrower age strata, spline analysis and individual covariate estimates were secondary, and the prediction-model analyses, the Youden-based cutoff and all sensitivity analyses were exploratory. No adjustment was made for multiple comparisons; *p* values from secondary and exploratory analyses are descriptive and are not considered confirmatory evidence.

The data-entry workbook is compatible with Microsoft Excel 2016 (Microsoft Corporation, Redmond, WA, USA) [38], and the data analysis was carried out in IBM SPSS Statistics for Windows, version 26.0 (IBM Corp., Armonk, NY, USA) [39] and R version 4.3.3 (R Foundation for Statistical Computing, Vienna, Austria) [40] with the packages sandwich 3.1.0, rms 6.7.1, pROC 1.18.5, mice 3.16.0 and car 3.1.2; the R code for the modified Poisson regression, sensitivity analyses and bootstrap procedures is available from the corresponding author (see Data Availability Statement).

### 2.10. Ethics

The study was approved by the Local Ethics Committee of Victor Babeș University of Medicine and Pharmacy Timișoara (approval no. 132583/2026, dated 22 September 2026); the approval and the waiver of informed consent covered the retrospective use of records from the entire study period (1 January 2022 to 31 May 2026). The study was conducted in accordance with the 2024 revision of the World Medical Association Declaration of Helsinki, the Council for International Organizations of Medical Sciences ethical guidelines, applicable institutional requirements and current publication-ethics recommendations [41,42,43]. The requirement for informed consent was waived by the Ethics Committee because of the retrospective design. All data were extracted, compiled and stored in accordance with the EU General Data Protection Regulation.

## 3. Results

### 3.1. Participants

Of 957 screened records, 96 were excluded as ineligible: 48 postmenopausal, 31 with an indication other than AUB and 17 pregnancy related. No screened record met the exclusion criteria of prior hysterectomy or known endometrial malignancy under treatment. Of 861 otherwise eligible women, 37 lacked adequate or available index histopathology, leaving 824 in the primary analysis (Figure 1). The analysed cohort comprised 361 women aged <45 and 463 aged ≥45 years. Reported yearly inclusion was 190 in 2022, 185 in 2023, 172 in 2024, 199 in 2025 and 78 during January–May 2026. Patient-level data for the 37 excluded women were not available in the analytic dataset, so they could not be separated into inadequate specimens and unavailable reports or described by age group and sampling method. These two mechanisms of missing outcome data may relate differently to age, endometrial characteristics and sampling technique, so the reported yields apply only to women with adequate and available histopathology. Because records were consolidated at patient level before screening, the number of repeat procedures was not recorded separately. In restricted scenarios in which none of the 37 had significant pathology, the crude RR would be 2.04 if all were aged <45 years and 1.71 if all were aged ≥45 years. Over the full range of possible outcomes, the crude RR would range from 0.80 (all 37 aged <45 years with pathology) to 2.83 (all aged ≥45 years with pathology); the direction of the association would reverse only if at least 25 of the 37 excluded women were younger than 45 years and had significant pathology, a frequency of about 68% compared with 9.8% among the analysed women. Complete and incomplete cases among the 824 analysed women are compared in Supplementary Table S6.

### 3.2. Participant Characteristics

Mean age was 45.0 years (SD 5.8), and mean BMI was 27.3 kg/m^2^ (SD 4.9). BMI was available for 807 women, of whom 242 (30.0%) met the predefined definition of obesity (BMI ≥ 30 kg/m^2^). Chronic anovulation was present in 194 women (23.5%), nulliparity in 175 (21.2%) and a high-risk bleeding pattern in 306 (37.1%). All 98 women with PCOS were also coded as chronically anovulatory. The primary outcome occurred in 9 of the 36 women with diabetes (25.0%) and EIN/carcinoma in 3 (8.3%); among women younger than 45 years, these numbers were 1 of 4 for each endpoint, and among those aged 45 years or older, 8 and 2 of 32. Baseline characteristics are summarised descriptively in Table 1. At least one of persistent AUB, failed medical treatment, obesity or chronic anovulation (including PCOS) was documented with similar frequency in women aged <45 years (291/361, 80.6%) and ≥45 years (375/463, 81.0%; *p* = 0.93), whereas a suspected polyp was more frequent at ≥45 years (21.0% vs. 12.7%; *p* = 0.002) (Supplementary Table S3). Among the 70 women younger than 45 years with none of these four characteristics, the broad outcome occurred in 4 (5.7%, 95% CI 2.2–13.8) and EIN/carcinoma in 2 (2.9%, 0.8–9.8); 43 of them had none of the characteristics listed in Supplementary Table S3, including intermenstrual or mixed bleeding and a suspected polyp (broad outcome 3/43, 7.0%, 2.4–18.6; EIN/carcinoma 1/43, 2.3%, 0.4–12.1).

### 3.3. Histopathological Yield

Significant endometrial pathology was identified in 81/824 women (9.8%; 95% CI 8.0–12.1): 52 had hyperplasia without atypia; 17 had EIN/atypical hyperplasia, and 12 had carcinoma. The clinically more consequential endpoint of EIN/atypical hyperplasia or carcinoma occurred in 29 women (3.5%; 95% CI 2.5–5.0). The full histopathological distribution is shown in Table 2, and the distribution by sampling method in Supplementary Table S7. A suspected polyp had been documented before 32/106 hysteroscopic biopsies (30.2%), compared with 38/200 curettage procedures (19.0%) and 73/518 Pipelle biopsies (14.1%), whereas benign polyps were found on histology with similar frequency (13.2%, 13.0% and 11.8%, respectively). If the 37 women without adequate or available histopathology are retained in the denominator as attempted sampling episodes, the yield was 81/861 (9.4%) for the broad outcome and 29/861 (3.4%) for EIN/carcinoma. Negative index histology was not verified by repeat sampling, hysterectomy or follow-up, so these figures are yields of detected pathology rather than estimates of disease prevalence.

### 3.4. Primary Age Comparison

Significant pathology occurred in 24/361 women aged <45 years (6.6%) and 57/463 aged ≥45 years (12.3%), an absolute difference of 5.7 percentage points (95% CI 1.6–9.6). The crude RR was 1.85 (95% CI 1.17–2.92; *p* = 0.008) in all 824 women and 1.82 (95% CI 1.14–2.90; *p* = 0.013) in the 776 complete cases, so the difference between the crude and adjusted estimates reflects covariate adjustment rather than the exclusion of incomplete cases. In the same complete cases, the adjusted RR from the modified Poisson model was 1.62 (95% CI 1.01–2.61; *p* = 0.047), and the complementary adjusted OR was 1.75 (95% CI 1.02–3.00; *p* = 0.042). These two measures estimate different quantities, and the adjusted RR is a conditional association within the sampled population rather than a causal effect of age. The adjusted estimates are compatible with anything from no association to a 2.6-fold higher yield at age ≥45 years; the association is therefore uncertain and should not be regarded as either established or absent.

EIN/atypical hyperplasia or carcinoma occurred in 4/361 women younger than 45 years (1.1%) and 25/463 aged 45 years or older (5.4%), an absolute difference of 4.3 percentage points (95% CI 1.9–6.8) and a crude RR of 4.87 (95% CI 1.71–13.88; *p* = 0.003). EIN/atypical hyperplasia and carcinoma are shown separately by age stratum in Table 3. Because only 29 EIN/carcinoma events occurred, this secondary estimate was not interpreted through a fully adjusted model. The following operating characteristics are conditional proportions among women already selected for sampling and do not describe the performance of an age-based biopsy policy: for EIN/carcinoma, the 45-year threshold had a conditional sensitivity of 86.2% (25/29; 95% CI 69.4–94.5), specificity of 44.9% (357/795; 41.5–48.4), positive predictive value of 5.4% (3.7–7.8) and negative predictive value of 98.9% (97.2–99.6).

### 3.5. Age–Yield Relationship and Model Performance

**Figure 2:**
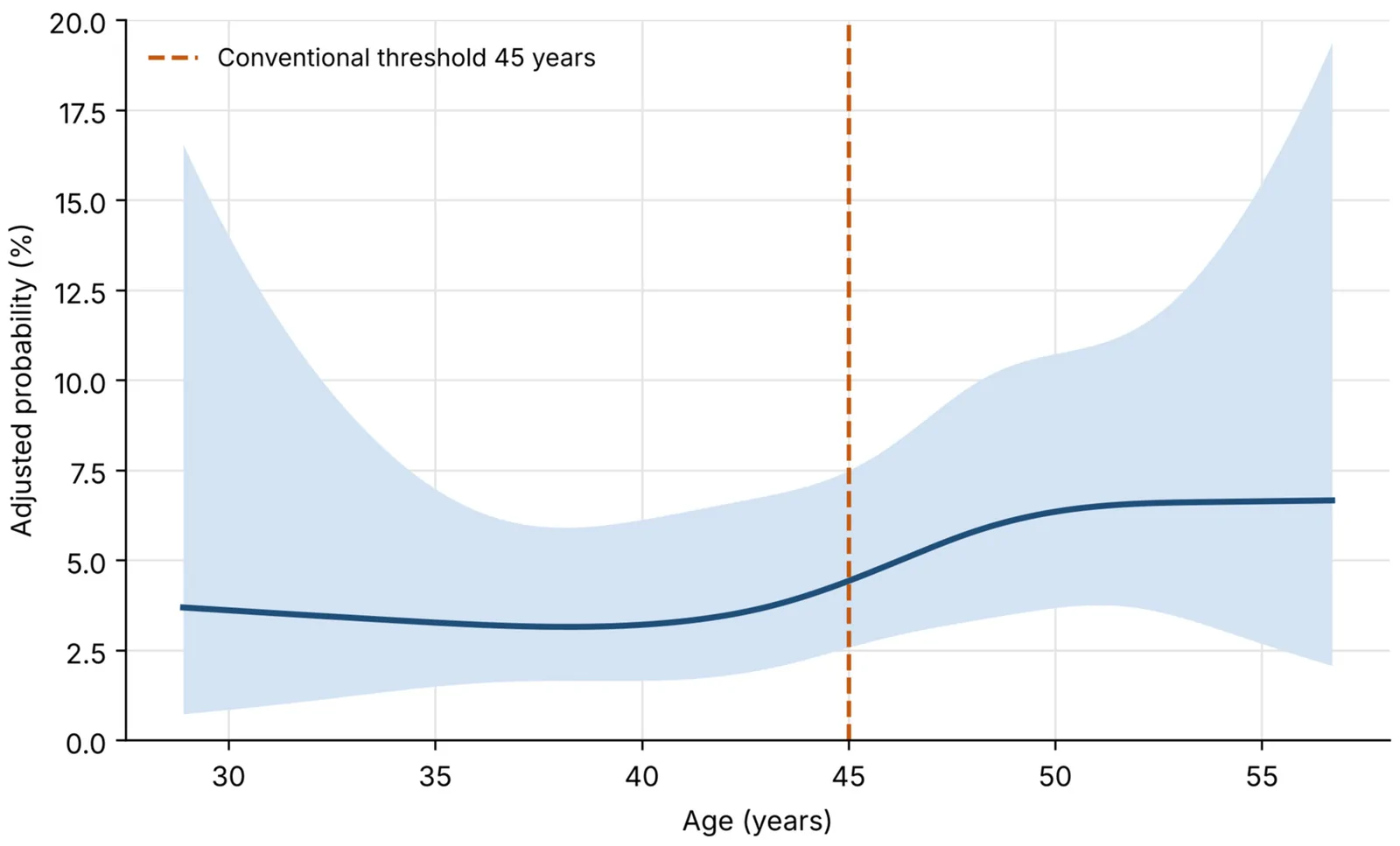
Adjusted association between age and significant endometrial pathology, estimated using multivariable logistic regression with age modelled as a restricted cubic spline. The solid line represents the predicted probability, the shaded area the 95% confidence interval, and the vertical dashed line the conventional 45-year threshold. Predictions refer to a single reference patient without obesity, diabetes, chronic anovulation, nulliparity or high-risk bleeding, with median endometrial thickness (9.2 mm) and Pipelle biopsy as the sampling method; they are conditional predictions for this covariate pattern and do not represent the average probability of pathology among all women of a given age, which is shown descriptively in Table 3. Wider confidence intervals at the age extremes reflect fewer observations. The curve is relatively flat below about 42 years and imprecise at the extremes, and it should not be read as evidence of a consistently monotonic relationship. The logistic model was used for this figure because it yields bounded predicted probabilities; the adjusted RR in Table 4 was estimated separately by modified Poisson regression.

Observed yield rose from 4.5% at age <40 to 8.3% at 40–44, 10.7% at 45–49 and 15.3% at ≥50 years (Table 3; Supplementary Figure S2). In the adjusted continuous-age analysis, the likelihood-ratio test did not detect departure from linearity (*p* = 0.709). This does not establish that the relationship is linear or exclude a clinically relevant change near 45 years; the fitted curve was relatively flat below about 42 years and rose gradually thereafter, without an abrupt change at 45 years, and its confidence band was wide at the extremes of the age distribution (Figure 2); neither this pattern nor the nonsignificant test establishes a consistently monotonic association.

**Table 4:** Modified Poisson model for significant endometrial pathology (complete cases, n = 776).

Variable	Adjusted RR	95% CI	*p* Value
Age ≥45 years	1.62	1.01–2.61	0.047
Obesity	1.30	0.85–2.00	0.223
Diabetes mellitus	2.00	1.02–3.94	0.044
Chronic anovulation	1.28	0.81–2.04	0.293
Nulliparity	2.07	1.35–3.16	<0.001
Intermenstrual or mixed bleeding	1.60	1.05–2.46	0.031
Endometrial thickness, per 1 mm	1.12	1.04–1.21	0.002
Hysteroscopic biopsy vs. D&C	0.58	0.27–1.24	0.159
Pipelle biopsy vs. D&C	0.95	0.58–1.56	0.835

RR, risk ratio; CI, confidence interval; D&C, dilatation and curettage. Endometrial thickness is interpreted per 1 mm increase. Obesity is defined as BMI ≥ 30 kg/m^2^. Covariates were defined before analysis. RRs are ratios of the proportions with detected pathology at index sampling. Covariate estimates are each adjusted for the other model terms and should not be interpreted as total effects of those factors.

Within the already sampled cohort, the ≥45-year cutoff had conditional sensitivity 70.4% (57/81; 95% CI 59.7–79.2), specificity 45.4% (337/743; 41.8–49.0), positive predictive value 12.3% (9.6–15.6) and negative predictive value 93.4% (90.3–95.5) for the broad outcome, corresponding to an AUC of 0.579 for the binary classifier. The exploratory Youden cutoff was 46.9 years (sensitivity 65.4%, specificity 57.3%) when estimated and assessed in all 824 women; because it was derived and evaluated in the same data, it is reported descriptively and is not proposed as a clinical threshold. With age entered as a continuous variable, age alone had an AUC of 0.612 (95% CI 0.549–0.676) in the full 824 and 0.604 (0.538–0.670) among the 776 complete cases (0.577, 0.521–0.632, with the binary ≥45-year term). The exploratory model restricted to pre-biopsy variables had an apparent AUC of 0.714 (0.651–0.776) on those same 776 cases (0.713, 0.650–0.777, when continuous age replaced the binary term). For the EIN/carcinoma endpoint, continuous age had an AUC of 0.713 (0.624–0.803) in the full cohort. After bootstrap internal validation with both models refitted in each resample, the principal like-for-like comparison (binary age in both models) gave optimism-corrected AUCs of 0.686 for the pre-biopsy model and 0.575 for age alone, a corrected difference of 0.111 (95% CI 0.060–0.196); with continuous age in both models, the corrected AUCs were 0.686 and 0.604 and the difference 0.082 (0.030–0.171). These are improvements in ranking within the sampled population and do not demonstrate clinical utility. The optimism-corrected calibration-in-the-large of the pre-biopsy model was 0.01 (approximate 95% interval −0.24 to 0.28) and its calibration slope 0.87 (0.65–1.15), indicating predictions that were correct on average but too extreme, consistent with overfitting; the calibration curve also deviated at predicted probabilities above about 0.20, where data were sparse (Supplementary Figure S3). Model performance is summarised in Table 5. The adjusted association model, including the sampling method, had an apparent AUC of 0.721.

### 3.6. Sensitivity Analyses

The age estimates from the sensitivity analyses are shown in Table 6. Across all specifications the adjusted RR for age ≥45 years ranged from 1.52 to 1.68. The lower confidence limit lay close to 1.00 under every variance estimator and covariate set: with HC3 standard errors the 95% CI was 1.00–2.63 (*p* = 0.051), with the bootstrap 1.01–2.79, without sampling method 0.99–2.59, and without endometrial thickness 1.04–2.71. Flexible modelling of BMI and endometrial thickness (1.64, 1.02–2.64), use of the recorded obesity indicator (1.61, 1.00–2.60), multiple imputation in all 824 women (1.66, 1.04–2.64), age >45 versus ≤45 years (1.67, 1.04–2.69) and classification of irregular bleeding as high risk (1.67, 1.03–2.68) gave similar estimates. Excluding women aged ≥50 years, which reduced the comparison to 45–49 versus <45 years and the events to 54, gave 1.52 (0.90–2.55). Variance inflation factors were all ≤1.08, and no single observation changed the age coefficient by more than 0.21 standard errors. The diabetes estimate was based on nine events among the 34 women with diabetes in the complete-case population; only one of these events occurred in a woman younger than 45 years (three women with diabetes in this age group had complete data), and the estimate should be interpreted with particular caution.

## 4. Discussion

### 4.1. Principal Findings

Among 824 premenopausal women already selected for sampling, EIN/carcinoma occurred in 29 (3.5%), 25 of them at age ≥45 years, and broad significant pathology occurred in 81 (9.8%), including 52 with hyperplasia without atypia. Broad-outcome yield was 6.6% below age 45 versus 12.3% at age ≥45. The adjusted association was imprecise (RR 1.62, 95% CI 1.01–2.61; *p* = 0.047), and no sharp age–yield discontinuity was detected. These findings describe a sampled cohort and cannot estimate the effect of applying a biopsy threshold to all women presenting with AUB.

Age alone had modest apparent discrimination on the 776 complete cases (AUC 0.604). An exploratory model using variables available before biopsy had higher discrimination (optimism-corrected AUC 0.686), but the internally validated gain over the age ≥45 rule (0.111, 95% CI 0.060–0.196) describes ranking within the sampled population, has not been confirmed externally and does not establish clinical utility. Diabetes, nulliparity, intermenstrual/mixed bleeding and endometrial thickness were associated with the broad outcome in the adjusted model, but these covariate estimates are imprecise, were not the main analysis and should not be read as confirmatory or as applying to EIN/carcinoma, which was too infrequent (29 events) for adjusted analysis.

The direction of several observed associations is compatible with the existing endometrial-risk literature [6,7,8,12,13,14,15]. Interpretation must nevertheless distinguish biological risk, clinician selection for sampling, and detection by different sampling methods.

### 4.2. Absolute Diagnostic Yield in Context

The broad primary-outcome yield of 9.8% should not be compared directly with population-level cancer risk. It included hyperplasia without atypia, which accounted for 52 of 81 events, and its denominator comprised women who had already crossed a clinical threshold for sampling. By contrast, Pennant and colleagues pooled studies of premenopausal women with AUB and estimated endometrial cancer at 0.33% and cancer or atypical hyperplasia at 1.31%, with lower cancer risk in heavy menstrual bleeding alone than in intermenstrual bleeding [12]. The 3.5% EIN/carcinoma frequency in our cohort is higher than that pooled estimate but remains plausible for a referred, procedurally selected population enriched for older age and additional risk factors [12,13,14,15].

Differences between published yields are expected because case mix and outcome definitions vary substantially. Some studies include all women presenting with AUB, whereas others include only those undergoing biopsy, hysteroscopy or surgery; age limits, menopausal definitions, referral setting and prevalence of obesity or PCOS also differ [12,13,14,15]. In a recent cohort restricted to sampled women aged 45 years or younger, atypical hyperplasia or endometrial cancer was found in 6.0%, with nulliparity, PCOS and multiple polyps associated with risk [13]. Giannella and colleagues likewise showed that combinations of clinical and sonographic factors may identify higher-risk premenopausal women more effectively than any isolated variable [14]. The IETA-1 cohort further demonstrates the value of structured symptom and ultrasound assessment in women with abnormal vaginal bleeding [15]. Comparisons with these studies should therefore be made cautiously because referral populations, outcome definitions and the criteria used to select women for biopsy differ.

The low absolute frequency of cancer remains clinically important. Endometrial cancer is often diagnosed after abnormal bleeding, and outcomes depend on tumour biology and stage, making timely evaluation of concerning symptoms essential [6]. At the same time, indiscriminate invasive testing exposes many women with benign disease to discomfort, anxiety, procedural complications, inadequate samples and additional investigations [11,16,17,18]. The central methodological and clinical problem is therefore not whether age matters but whether age improves decision-making enough to justify biopsy when considered alongside the patient’s other risk indicators.

The bleeding burden in this cohort was also reflected by a mean pre-sampling haemoglobin of 11.4 g/dL, which did not differ between age groups. In a retrospective series of 543 women undergoing hysteroscopy for AUB, Jobda et al. found that post-procedural haemoglobin varied with the FIGO-based aetiology of bleeding and with age; however, that cohort included both pre- and postmenopausal women and is therefore not directly comparable with ours [44].

### 4.3. Meaning of the 45-Year Threshold

The conventional threshold has practical value because it is simple, memorable and sensitive to the increasing incidence of important endometrial pathology with age. ACOG explicitly uses age older than 45 years as a first-line sampling indication in AUB, while extending sampling to younger women with unopposed oestrogen exposure, persistent bleeding or failed treatment [9,10]. NICE takes a more explicitly risk-based approach in heavy menstrual bleeding and recommends considering biopsy at hysteroscopy in selected high-risk women rather than offering blind biopsy routinely [11].

Within this sampled cohort, the age cutoff identified 57/81 broad-outcome events, but 24/81 occurred below it. Among women without the broad outcome, 406/743 were aged ≥45. These conditional operating characteristics cannot be interpreted as the sensitivity or specificity of an age-based biopsy policy among all women presenting with AUB because pathology is unknown for those who were never sampled. For EIN/carcinoma, 25/29 sampled cases occurred at age ≥45; this more consequential endpoint was uncommon, and its estimates are imprecise.

The exploratory Youden-optimal cutoff of 46.9 years should not be regarded as a preferable clinical threshold because it did not address selection bias or within-sample optimism. A data-derived threshold chosen and evaluated in the same cohort is vulnerable to optimism and sampling variability, and Youden’s index gives equal weight to sensitivity and specificity even when the clinical consequences of missed EIN/cancer and unnecessary biopsy are not equivalent [15,45]. Moreover, the spline analysis was compatible with a gradual age–yield relationship rather than a discontinuity at 45 years, although a nonsignificant test for nonlinearity cannot establish the shape of the relationship. A threshold converts a continuous gradient into an actionable rule; it should not be interpreted as the day on which endometrial biology changes.

Menopausal status and chronological age must also remain conceptually separate. The cohort required documented premenopausal or perimenopausal status, so women aged 50 years or older could remain eligible if they had not completed 12 months of non-pathological amenorrhoea. Conversely, ovarian dysfunction and anovulatory exposure may occur at younger ages. FIGO terminology, endometrial cancer biology and guideline recommendations all support assessing the bleeding phenotype and hormonal risk context rather than treating age as a surrogate diagnosis [1,2,6,9,10,11]. When women aged ≥50 years were excluded, the RR for 45–49 versus <45 years was 1.52 (95% CI 0.90–2.55): the estimate remained positive but was smaller and less precise, so part of the overall gradient was carried by the oldest stratum, in which menopausal classification is least certain (Table 6). This restricted analysis is itself imprecise and cannot resolve possible misclassification of menopausal status. Because cycle status was not recorded separately, a restriction to women with documented ongoing cycles was not possible. Nothing in these results supports a secular claim that menopause is occurring earlier; that would require a different, population-based longitudinal design.

### 4.4. Clinical Predictors and Biological Plausibility

The following associations refer to the broad outcome, of which hyperplasia without atypia accounted for 52 of 81 events and cannot be attributed to EIN or carcinoma. The adjusted association with diabetes is biologically and epidemiologically plausible, but it was based on only nine events among the 36 women with diabetes (one event among the 4 women with diabetes younger than 45 years and eight among the 32 aged 45 years or older), its confidence interval was wide, and it may be unstable. Mechanisms proposed in previous research include hyperinsulinaemia, insulin-like growth factor signalling, chronic inflammation and frequent coexistence with obesity [6,7], which apply mainly to type 2 diabetes. Diabetes type could not be distinguished here; these were not measured here, and the present data cannot attribute the association to any of them. Obesity is one of the most consistently documented modifiable risk factors for endometrial cancer, with risk increasing as BMI rises [6,7]. In this model, the binary obesity term did not remain independently associated with the outcome after adjustment; that should not be interpreted as evidence against the established association. Correlation with diabetes, anovulation and endometrial thickness, loss of information from dichotomising BMI (continuous BMI gave an RR of 1.06 per 5 kg/m^2^, 95% CI 0.85–1.31), limited event numbers and selection of already-sampled women can attenuate an adjusted coefficient [6,7,8,14]. Because endometrial thickness may lie on the pathway between metabolic risk and pathology, the coefficients for obesity and diabetes describe associations conditional on thickness rather than total effects [29].

Nulliparity may reflect the absence of pregnancy-associated progesterone exposure, but in women of reproductive age it may equally be a marker of infertility, anovulation, PCOS or other endocrine disorders; infertility history was not available, so these explanations could not be distinguished. The association observed here is consistent with premenopausal risk-factor studies, including the strong nulliparity signal reported in younger sampled women [8,13,14]. Chronic anovulation and PCOS are clinically important because prolonged cycles can produce extended periods of unopposed endometrial stimulation; however, their documentation in routine records is often incomplete, and a nonsignificant adjusted estimate may represent measurement error; limited power; or overlap with BMI, diabetes and bleeding pattern rather than biological absence [6,7,8,13,14]. These biological explanations are drawn from previous research and were not tested directly in this study.

The combined intermenstrual/mixed bleeding indicator was associated with pathology. This agrees with the pooled evidence that cancer risk is higher among premenopausal women with intermenstrual bleeding than among those with heavy menstrual bleeding alone and with guideline emphasis on persistent intermenstrual or irregular bleeding [11,12]. Endometrial thickness also contributed to the clinical model, but its effect should be interpreted cautiously: before menopause, thickness varies with cycle phase, ovulatory regularity, medication and focal lesions, and no single cutoff has achieved universal acceptance [11,14,19]. Because cycle phase and hormonal treatment at the time of ultrasonography were not available, measurement variability may have attenuated this association, or treatment may have confounded it. Ultrasound is therefore most useful as one component of structured assessment, including endometrial morphology and intracavitary lesions, rather than as an autonomous biopsy rule [11,14,15,19].

### 4.5. Sampling Method and Outcome Ascertainment

The diagnostic pathway influences the observed yield. Office aspiration biopsy is convenient and often accurate for diffuse endometrial disease, whereas hysteroscopy enables visualisation and targeted sampling of focal abnormalities; dilatation and curettage obtains more tissue but is more invasive and still may miss focal lesions when performed blindly [11,16,17,18]. A systematic review of hysteroscopy in women with AUB found high diagnostic accuracy for endometrial cancer but only moderate accuracy for endometrial disease overall (cancer or hyperplasia) [46]. Systematic reviews of blind sampling show that endometrial sampling can have high specificity and useful sensitivity for cancer, but performance varies with reference standard, disease spectrum, device and sample adequacy [16,17,18]. Failed or insufficient samples are not neutral observations: they may lead to repeat biopsy, hysteroscopy or surveillance and can introduce verification bias if excluded from the primary analysis [17,18].

Our association analysis required an adequate histopathological result and adjusted for sampling method, which supports a clear diagnostic-yield denominator but does not eliminate method-related selection. Clinicians may preferentially choose hysteroscopy for suspected polyps or focal lesions and aspiration biopsy for diffuse endometrial assessment; consequently, the sampling method can be both a measurement characteristic and a marker of pre-test suspicion. The lower adjusted RR for hysteroscopic biopsy than for dilatation and curettage (0.58, 95% CI 0.27–1.23) should be read in this light. A suspected polyp had been documented before 30.2% of hysteroscopic biopsies, compared with 19.0% of curettage procedures and 14.1% of Pipelle biopsies, whereas benign polyps were found on histology with similar frequency across methods (Supplementary Table S7). Hysteroscopy may therefore have been used more often for suspected focal lesions, which are mostly benign. Differences in case mix are one possible explanation, but because the reasons for choosing each technique and the frequency of inadequate specimens by technique were not recorded, case mix cannot be distinguished from differences in diagnostic performance, and no conclusion about comparative accuracy can be drawn. Omitting the sampling method from the model changed the age estimate only slightly, from 1.62 to 1.60 (Table 6). Future studies should report inadequate samples separately; describe subsequent investigations; and, if feasible, compare index histology with hysterectomy or follow-up pathology rather than treating every negative sample as definitive [11,16,17,18].

### 4.6. Model Performance and Clinical Interpretation

The exploratory pre-biopsy model improved apparent discrimination from AUC 0.604 for age alone to 0.714 on the same 776 complete cases. The sampling method was excluded because it is chosen in the diagnostic pathway and is not an appropriate predictor for a decision to biopsy. An AUC of 0.714 describes ranking in this selected cohort; the bootstrap optimism-corrected estimate was 0.686, and the optimism-corrected gain over the age ≥45 rule was 0.111 (95% CI 0.060–0.196). Calibration-in-the-large was close to zero (0.01), but the corrected calibration slope of 0.87 (approximate 95% interval 0.65–1.15) indicates overfitting, with predicted probabilities that were too extreme, and the calibration curve deviated at higher predicted probabilities, where data were sparse; calibration assessed in the development sample gives limited assurance about performance in future patients. Several further cautions apply. Better discrimination within the sampled population does not establish clinical utility: the model was developed only among women already selected for biopsy. Its performance among unsampled women is unknown, and better ranking of sampled women does not show that using the model would improve decisions about who should be sampled. With 76 events, it is also prone to overfitting. The model is therefore not suitable for guiding biopsy decisions; development in an unselected AUB population, external validation, calibration assessment and decision-analytic evaluation, such as decision-curve analysis [37], would be required before any clinical use.

### 4.7. Strengths and Limitations

This study has several strengths. Consecutive inclusion establishes an explicit denominator of sampled premenopausal women and reduces investigator-driven case selection; age was analysed dichotomously, in clinically interpretable strata and continuously. Both RR and absolute yield were reported, and the EIN/carcinoma endpoint was separated from the broader composite. Transparent participant flow, missing-data denominators and reporting matrices follow STROBE and RECORD recommendations for observational studies based on routine data [20,21]. Modified Poisson regression provides an interpretable adjusted RR for a binary outcome, and spline modelling avoids assuming that a chosen age cutoff is the only relevant representation of age [28,30].

Several limitations are equally important. First, this is a cohort of sampled women, not a population cohort of all women with AUB. Referral and sampling were determined by clinical judgment and guideline-influenced practice, creating selection bias and confounding by indication. Because the decision to sample depends on age and on factors associated with pathology, such as bleeding pattern, metabolic risk and imaging findings, restricting the analysis to sampled women conditions on a common consequence of age and pathology, this can induce collider (selection) bias that multivariable adjustment cannot remove [47]. If younger women were sampled mainly when additional risk factors were present, their yield would be inflated and the age association attenuated; if older women were sampled more liberally on the basis of age alone, the yield at ≥45 years would be diluted, again attenuating the association. Age-related differences in sampling technique and detection could bias the estimate in either direction. The magnitude of these effects cannot be estimated from the available data. Selected guideline-related characteristics were documented with similar frequency in both age groups (Supplementary Table S3), but because only sampled women were observed, this cannot show that the probability of being sampled was similar across ages and does not exclude differential selection. The observed yield cannot be interpreted as disease prevalence among all premenopausal women with AUB, nor can the study determine what happened to otherwise eligible women who were not sampled. Second, a single-centre case mix, local referral network and distribution of sampling procedures may limit transportability.

Third, routinely collected variables may be incomplete or inconsistently defined. Menopausal status, cycle timing, bleeding volume, chronic anovulation, treatment failure and PCOS can be misclassified, while BMI, medication exposure and family history may be missing not at random. Current or recent hormonal treatment, infertility history and hereditary predisposition, such as Lynch syndrome, were not available in the dataset; because these factors influence both histology and sampling decisions, residual confounding is possible, particularly for the associations involving nulliparity and chronic anovulation. Cycle phase at ultrasonography, the specific indication for sampling and individual sampling dates were not available; menopausal status was based on clinical documentation that could not be verified, and agreement between data extractors was not quantified, although discrepancies were resolved by consensus. Patient-level data on the 37 women excluded for inadequate or unavailable histopathology were not retained, so their characteristics could not be compared with those of the included women, although the scenario analysis in Section 3.1 shows that the direction of the crude association would reverse only under extreme assumptions. Complete-case analysis is unbiased only under restrictive assumptions and can reduce precision, although multiple imputation gave a similar estimate (RR 1.66). Fourth, pathology was taken from routine reports without central blinded review, and diagnostic verification differed by sampling method; focal disease and inadequate samples may therefore be misclassified [16,17,18]. The distinction between disordered proliferative endometrium and hyperplasia without atypia is subject to known inter-observer variability, and negative index histology was not verified by repeat sampling, hysterectomy or follow-up; the absence of pathology in women with negative findings therefore cannot be established.

Fifth, the composite primary outcome combines hyperplasia without atypia with EIN and carcinoma. This increased statistical power but groups conditions with different natural histories and management consequences. The EIN/carcinoma endpoint was clinically more specific but occurred only 29 times, precluding a stable fully adjusted model and producing imprecise estimates.

Sixth, only 76 events contributed to a nine-parameter complete-case association model (approximately 8.4 events per parameter), creating instability and imprecision. The adjusted age RR has a lower confidence limit of 1.01, and its *p* value of 0.047 is sensitive to modelling details, such as the choice of robust variance estimator (Table 6). The exploratory pre-biopsy model has no external validation, and even its internally validated performance applies only to women already selected for sampling. The age cutoff derived with Youden’s index was selected and assessed in the same sample.

### 4.8. Clinical and Research Implications

Clinically, these data reinforce the need to consider age within a broader risk assessment, as current ACOG and NICE recommendations already do [9,10,11]. They do not establish a new biopsy threshold: patients not sampled were outside the analysis, and a validated rule would need outcomes ascertained across a clinically relevant AUB population. Decisions for younger patients with obesity, diabetes, chronic anovulation, persistent irregular/intermenstrual bleeding, failed treatment or concerning imaging should continue to follow clinical guidance and individual assessment.

Future work should prospectively or retrospectively assemble a consecutive population of women presenting with AUB, document who was and was not sampled and ascertain important outcomes with adequate follow-up.

## 5. Conclusions

Among premenopausal women already selected for endometrial sampling because of abnormal uterine bleeding, the yield of significant endometrial pathology increased across age strata, and 25 of 29 cases of EIN/atypical hyperplasia or carcinoma occurred at age ≥45 years, although this endpoint was uncommon. The adjusted comparison for the broad outcome at age 45 years was imprecise, and age alone had modest discrimination. The exploratory pre-biopsy model showed higher apparent and optimism-corrected discrimination than age alone in the same complete-case cohort, but it has not been externally validated, and its usefulness for biopsy decisions is unknown. These findings describe the yield of detected pathology among sampled women and cannot establish an age-only biopsy rule for all women presenting with abnormal uterine bleeding.

## Figures and Tables

**Table 1:** Baseline characteristics of the cohort by primary age group.

Characteristic	Overall	<45 Years	≥45 Years
Age, years	45.0 (5.8)	39.6 (3.9)	49.3 (2.7)
BMI, kg/m^2^	27.3 (4.9)	26.8 (4.6)	27.7 (5.0)
Obesity, BMI ≥ 30 kg/m^2^	242/807 (30.0%)	92/356 (25.8%)	150/451 (33.3%)
Nulliparity	175 (21.2%)	83 (23.0%)	92 (19.9%)
Diabetes mellitus	36 (4.4%)	4 (1.1%)	32 (6.9%)
Hypertension	68 (8.3%)	18 (5.0%)	50 (10.8%)
PCOS	98 (11.9%)	49 (13.6%)	49 (10.6%)
Chronic anovulation	194 (23.5%)	87 (24.1%)	107 (23.1%)
Persistent AUB	397 (48.2%)	171 (47.4%)	226 (48.8%)
Intermenstrual or mixed bleeding	306 (37.1%)	128 (35.5%)	178 (38.4%)
Endometrial thickness, mm	9.3 (2.9)	9.0 (2.9)	9.5 (2.8)
Haemoglobin, g/dL	11.4 (1.3)	11.4 (1.3)	11.4 (1.3)
Suspected endometrial polyp	143 (17.4%)	46 (12.7%)	97 (21.0%)
Tamoxifen exposure	3 (0.4%)	0 (0.0%)	3 (0.6%)
Current smoking	145 (17.6%)	66 (18.3%)	79 (17.1%)
Failed medical treatment	385 (46.7%)	167 (46.3%)	218 (47.1%)
Uterine fibroid on ultrasound	246 (29.9%)	91 (25.2%)	155 (33.5%)
Pipelle biopsy	518 (62.9%)	248 (68.7%)	270 (58.3%)
Dilatation and curettage	200 (24.3%)	79 (21.9%)	121 (26.1%)
Hysteroscopic biopsy	106 (12.9%)	34 (9.4%)	72 (15.6%)

Values are mean (standard deviation) or n (%), unless otherwise indicated. BMI was available for 807 participants (356 aged <45 and 451 aged ≥45 years), endometrial thickness for 793 (347 and 446) and haemoglobin for 812 (356 and 456); means for these variables are calculated among participants with available data. Percentages for obesity are calculated among participants with available BMI. AUB, abnormal uterine bleeding; BMI, body mass index; PCOS, polycystic ovary syndrome.

**Table 2:** Histopathological findings from the index endometrial sampling procedure.

Histopathology	n	%
Proliferative endometrium	234	28.4%
Secretory endometrium	151	18.3%
Disordered proliferative endometrium	150	18.2%
Benign endometrial polyp	101	12.3%
Atrophic or inactive endometrium	67	8.1%
Hyperplasia without atypia	52	6.3%
Other benign histology	40	4.9%
EIN or atypical hyperplasia	17	2.1%
Endometrial carcinoma	12	1.5%

**Table 3:** The diagnostic yield by predefined age stratum, with the components of the broad outcome.

Age, Years	N	Broad Outcome, n (%)	95% CI, %	HWA, n	EIN/AH, n	Cancer, n	EIN or Cancer, n (%)	95% CI, %
<40	155	7 (4.5%)	2.2–9.0	6	0	1	1 (0.6%)	0.1–3.6
40–44	206	17 (8.3%)	5.2–12.8	14	3	0	3 (1.5%)	0.5–4.2
45–49	300	32 (10.7%)	7.7–14.7	20	7	5	12 (4.0%)	2.3–6.9
≥50	163	25 (15.3%)	10.6–21.7	12	7	6	13 (8.0%)	4.7–13.2
Total	824	81 (9.8%)	8.0–12.1	52	17	12	29 (3.5%)	2.5–5.0

Broad outcome: hyperplasia without atypia (HWA), EIN/atypical hyperplasia (AH) or carcinoma (cancer). 95% CIs are Wilson score intervals. Estimates in the strata below 45 years are based on few events and are highly imprecise. EIN, endometrial intraepithelial neoplasia.

**Table 5:** Discrimination and calibration of the age-only and exploratory pre-biopsy models.

Model (Cohort)	Age Coding	Apparent AUC (95% CI)	Optimism-Corrected AUC	Calibration Intercept	Calibration Slope
Age only (n = 824)	Continuous	0.612 (0.549–0.676)	—	—	—
Age only (n = 824)	≥45 years	0.579 (0.525–0.632)	—	—	—
Age only (n = 776)	Continuous	0.604 (0.538–0.670)	0.604	—	—
Age only (n = 776)	≥45 years	0.577 (0.521–0.632)	0.575	—	—
Pre-biopsy model (n = 776)	≥45 years	0.714 (0.651–0.776)	0.686	0.01 (−0.24 to 0.28)	0.87 (0.65–1.15)
Pre-biopsy model (n = 776)	Continuous	0.713 (0.650–0.777)	0.686	0.01 (−0.23 to 0.27)	0.87 (0.64–1.19)
Difference, both with binary age (principal)	≥45 years	0.137	0.111 (0.060–0.196) †	—	—
Difference, both with continuous age	Continuous	0.109	0.082 (0.030–0.171) †	—	—

AUC, area under the receiver operating characteristic curve. Apparent AUC confidence intervals by the DeLong method. Optimism correction and calibration measures from 1500 bootstrap resamples with the model refitted in each resample; calibration intercept and slope values of 0 and 1 indicate ideal calibration. † Optimism-corrected difference with both models refitted in each resample; location-shifted bootstrap 95% CI [35]. Calibration intervals are the 2.5th–97.5th percentiles of the values obtained when bootstrap-refitted models were applied to the original data. Obesity is defined as BMI ≥ 30 kg/m^2^.

**Table 6:** Sensitivity analyses for the association between age ≥45 years and significant endometrial pathology.

Analysis	n	Events	RR or OR for Age ≥45 Years (95% CI)
Crude RR, all women	824	81	1.85 (1.17–2.92)
Crude RR, complete cases	776	76	1.82 (1.14–2.90)
Main adjusted model, HC0 robust SE	776	76	1.62 (1.01–2.61)
Main adjusted model, HC3 robust SE	776	76	1.62 (1.00–2.63)
Main adjusted model, bootstrap percentile CI	776	76	1.62 (1.01–2.79)
Without endometrial thickness	776	76	1.68 (1.04–2.71)
Without sampling method	776	76	1.60 (0.99–2.59)
Without endometrial thickness and sampling method	776	76	1.67 (1.03–2.71)
Continuous BMI and spline for endometrial thickness	776	76	1.64 (1.02–2.64)
Recorded obesity indicator instead of BMI ≥ 30 kg/m^2^	776	76	1.61 (1.00–2.60)
Multiple imputation of missing BMI and thickness	824	81	1.66 (1.04–2.64)
Excluding women aged ≥50 years	622	54	1.52 (0.90–2.55)
Age >45 versus ≤45 years	776	76	1.67 (1.04–2.69)
Irregular bleeding also classified as high risk	776	76	1.67 (1.03–2.68)
Logistic regression, adjusted OR	776	76	1.75 (1.02–3.00)

All adjusted models other than the logistic model are modified Poisson models with HC0 robust standard errors and the covariates listed in Table 4, unless stated otherwise. In the analysis excluding women aged ≥50 years, the comparison is 45–49 versus <45 years. Analyses restricted to women with documented ongoing cycles or adjusted for calendar year could not be performed because these variables were not available. All sensitivity analyses were post hoc. BMI, body mass index; CI, confidence interval; OR, odds ratio; RR, risk ratio; SE, standard error.

## Data Availability

Aggregate data supporting the findings are presented in the article and Supplementary Materials. The de-identified patient-level dataset is not publicly available because of institutional data-protection restrictions and the conditions of the Ethics Committee approval; they are available from the corresponding author upon reasonable request, subject to approval by the Ethics Committee of Victor Babeș University of Medicine and Pharmacy Timișoara and in compliance with Regulation (EU) 2016/679.

## Supplementary Materials

The following supporting information can be downloaded at https://www.mdpi.com/article/10.3390/jcm15218523/s1, Supplementary Table S1: STROBE reporting matrix; Supplementary Table S2: RECORD extension reporting matrix; Supplementary Table S3: Selected guideline-related characteristics by age group; Supplementary Table S4: Operational coding of histopathology outcomes; Supplementary Table S5: The specification of the exploratory pre-biopsy model; Supplementary Table S6: The comparison of complete and incomplete cases; Supplementary Table S7: Histopathology and suspected polyps by sampling method; Supplementary Figure S1: The assumed causal structure; Supplementary Figure S2: Age-stratified yield of both endpoints; Supplementary Figure S3: The calibration plot of the pre-biopsy model.

## References

[1] Jain V., Munro M.G., Critchley H.O.D. (2023). Contemporary evaluation of women and girls with abnormal uterine bleeding: FIGO Systems 1 and 2. Int. J. Gynaecol. Obstet..

[2] Munro M.G., Critchley H.O.D., Fraser I.S., FIGO Menstrual Disorders Committee (2018). The two FIGO systems for normal and abnormal uterine bleeding symptoms and classification of causes of abnormal uterine bleeding in the reproductive years: 2018 revisions. Int. J. Gynaecol. Obstet..

[3] Liu Z., Doan Q.V., Blumenthal P., Dubois R.W. (2007). A systematic review evaluating health-related quality of life, work impairment, and health-care costs and utilization in abnormal uterine bleeding. Value Health.

[4] Schoep M.E., Adang E.M.M., Maas J.W.M., De Bie B., Aarts J.W.M., Nieboer T.E. (2019). Productivity loss due to menstruation-related symptoms: A nationwide cross-sectional survey among 32,748 women. BMJ Open.

[5] Henry C., Ekeroma A., Filoche S. (2020). Barriers to seeking consultation for abnormal uterine bleeding: Systematic review of qualitative research. BMC Womens Health.

[6] Crosbie E.J., Kitson S.J., McAlpine J.N., Mukhopadhyay A., Powell M.E., Singh N. (2022). Endometrial cancer. Lancet.

[7] Wise M.R., Jordan V., Lagas A., Showell M., Wong N., Lensen S., Farquhar F.C.M. (2016). Obesity and endometrial hyperplasia and cancer in premenopausal women: A systematic review. Am. J. Obstet. Gynecol..

[8] Farquhar C.M., Lethaby A., Sowter M., Verry J., Baranyai J. (1999). An evaluation of risk factors for endometrial hyperplasia in premenopausal women with abnormal menstrual bleeding. Am. J. Obstet. Gynecol..

[9] American College of Obstetricians and Gynecologists (2012). Practice Bulletin No. 128: Diagnosis of abnormal uterine bleeding in reproductive-aged women. Obstet. Gynecol..

[10] American College of Obstetricians and Gynecologists (2013). Committee Opinion No. 557: Management of acute abnormal uterine bleeding in nonpregnant reproductive-aged women. Obstet. Gynecol..

[11] National Institute for Health and Care Excellence Heavy Menstrual Bleeding: Assessment and Management. NICE Guideline NG88. Updated 24 May 2021. https://www.nice.org.uk/guidance/ng88.

[12] Pennant M.E., Mehta R., Moody P., Hackett G., Prentice A., Sharp S.J., Lakshman R. (2017). Premenopausal abnormal uterine bleeding and risk of endometrial cancer. BJOG Int. J. Obstet. Gynaecol..

[13] Jang S., Hwang S.O. (2024). Risk factors for endometrial hyperplasia or cancer in premenopausal women aged 45 years or younger. Eur. J. Obstet. Gynecol. Reprod. Biol..

[14] Giannella L., Cerami L.B., Setti T., Bergamini E., Boselli F. (2019). Prediction of endometrial hyperplasia and cancer among premenopausal women with abnormal uterine bleeding. Biomed. Res. Int..

[15] Verbakel J.Y., Mascilini F., Wynants L., Epstein E., De Cock B., Pascual M.A., Leone F.P.G., Sladkevicius P., Alcazar J.L., Van Pachterbeke C. (2022). Risk assessment for endometrial cancer in women with abnormal vaginal bleeding: Results from the prospective IETA-1 cohort study. Int. J. Gynaecol. Obstet..

[16] Sakna N.A., Elgendi M., Salama M.H., Zeinhom A., Labib S., Nabhan A.F. (2023). Diagnostic accuracy of endometrial sampling tests for detecting endometrial cancer: A systematic review and meta-analysis. BMJ Open.

[17] Dijkhuizen F.P.H.L.J., Mol B.W.J., Brölmann H.A.M., Heintz A.P.M. (2000). The accuracy of endometrial sampling in the diagnosis of patients with endometrial carcinoma and hyperplasia: A meta-analysis. Cancer.

[18] van Hanegem N., Prins M.M.C., Bongers M.Y., Opmeer B.C., Sahota D.S., Mol B.W.J., Timmermans A. (2016). The accuracy of endometrial sampling in women with postmenopausal bleeding: A systematic review and meta-analysis. Eur. J. Obstet. Gynecol. Reprod. Biol..

[19] Giri S.K., Nayak B.L., Mohapatra J. (2021). Thickened endometrium: When to intervene? A clinical conundrum. J. Obstet. Gynaecol. India.

[20] von Elm E., Altman D.G., Egger M., Pocock S.J., Gøtzsche P.C., Vandenbroucke J.P., STROBE Initiative (2008). The Strengthening the Reporting of Observational Studies in Epidemiology statement: Guidelines for reporting observational studies. J. Clin. Epidemiol..

[21] Benchimol E.I., Smeeth L., Guttmann A., Harron K., Moher D., Petersen I., Sørensen H.T., von Elm E., Langan S.M., RECORD Working Committee (2015). The REporting of studies Conducted using Observational Routinely-collected health Data (RECORD) statement. PLoS Med..

[22] Royal College of Obstetricians and Gynaecologists, British Society for Gynaecological Endoscopy, British Gynaecological Cancer Society (2020). Joint Guidance for the Management of Abnormal Uterine Bleeding in the Evolving Coronavirus (COVID-19) Pandemic.

[23] WHO Classification of Tumours Editorial Board (2020). Female Genital Tumours. WHO Classification of Tumours.

[24] European Parliament and Council of the European Union (2016). Regulation (EU) 2016/679 of 27 April 2016 on the protection of natural persons with regard to the processing of personal data and on the free movement of such data. Off. J. Eur. Union.

[25] Parliament of Romania (2018). Law No. 190/2018 on Measures Implementing Regulation (EU) 2016/679.

[26] Peduzzi P., Concato J., Kemper E., Holford T.R., Feinstein A.R. (1996). A simulation study of the number of events per variable in logistic regression analysis. J. Clin. Epidemiol..

[27] Newcombe R.G. (1998). Interval estimation for the difference between independent proportions: Comparison of eleven methods. Stat. Med..

[28] Zou G. (2004). A modified Poisson regression approach to prospective studies with binary data. Am. J. Epidemiol..

[29] Westreich D., Greenland S. (2013). The Table 2 fallacy: Presenting and interpreting confounder and modifier coefficients. Am. J. Epidemiol..

[30] Desquilbet L., Mariotti F. (2010). Dose-response analyses using restricted cubic spline functions in public health research. Stat. Med..

[31] DeLong E.R., DeLong D.M., Clarke-Pearson D.L. (1988). Comparing the areas under two or more correlated receiver operating characteristic curves: A nonparametric approach. Biometrics.

[32] Sterne J.A.C., White I.R., Carlin J.B., Spratt M., Royston P., Kenward M.G., Wood A.M., Carpenter J.R. (2009). Multiple imputation for missing data in epidemiological and clinical research: Potential and pitfalls. BMJ.

[33] Long J.S., Ervin L.H. (2000). Using heteroscedasticity consistent standard errors in the linear regression model. Am. Stat..

[34] Harrell F.E., Lee K.L., Mark D.B. (1996). Multivariable prognostic models: Issues in developing models, evaluating assumptions and adequacy, and measuring and reducing errors. Stat. Med..

[35] Noma H., Shinozaki T., Iba K., Teramukai S., Furukawa T.A. (2021). Confidence intervals of prediction accuracy measures for multivariable prediction models based on the bootstrap-based optimism correction methods. Stat. Med..

[36] Van Calster B., McLernon D.J., van Smeden M., Wynants L., Steyerberg E.W. (2019). Calibration: The Achilles heel of predictive analytics. BMC Med..

[37] Vickers A.J., Elkin E.B. (2006). Decision curve analysis: A novel method for evaluating prediction models. Med. Decis. Mak..

[38] Microsoft Corporation (2016). Microsoft Excel 2016.

[39] IBM Corp (2019). IBM SPSS Statistics for Windows, Version 26.0.

[40] R Core Team (2024). R: A Language and Environment for Statistical Computing.

[41] World Medical Association (2025). World Medical Association Declaration of Helsinki: Ethical principles for medical research involving human participants. JAMA.

[42] Council for International Organizations of Medical Sciences (2016). International Ethical Guidelines for Health-Related Research Involving Humans.

[43] International Committee of Medical Journal Editors (2025). Recommendations for the Conduct, Reporting, Editing, and Publication of Scholarly Work in Medical Journals. https://www.icmje.org/recommendations/.

[44] Jobda K., Szeszko Ł., Wróbel G., Głuchowska M., Krupińska J., Szeszko A., Makaruk B., Oszukowski P., Zieliński P. (2022). Hemoglobin levels and platelet counts after hysteroscopy due to abnormal uterine bleeding. Diagnostics.

[45] Youden W.J. (1950). Index for rating diagnostic tests. Cancer.

[46] Clark T.J., Voit D., Gupta J.K., Hyde C., Song F., Khan K.S. (2002). Accuracy of hysteroscopy in the diagnosis of endometrial cancer and hyperplasia: A systematic quantitative review. JAMA.

[47] Hernán M.A., Hernández-Díaz S., Robins J.M. (2004). A structural approach to selection bias. Epidemiology.

