# Supplementary material for: Diagnostic Yield of Endometrial Sampling by Age in Premenopausal Women with Abnormal Uterine Bleeding: A Retrospective Cross-Sectional Study
Source: J Clin Med. 2026 Nov 2;15(21):8523. doi: 10.3390/jcm15218523 (PMC13764509; doi:10.3390/jcm15218523)
Supplement: Supplementary file 1 [file jcm-15-08523-s001.zip › jcm-4642815-supplementary.pdf]

Supplementary Tables

Diagnostic Yield of Endometrial Sampling by Age in Premenopausal Women with Abnormal Uterine Bleeding

S1 maps STROBE items, S2 maps RECORD items, and S4 defines histopathology outcome coding. Locations refer to the accompanying manuscript.

Supplementary Table S1. STROBE reporting matrix

Item	Reporting domain	Location and implementation
1	Title and abstract	Design (retrospective cross-sectional) identified in the title; structured abstract reports methods, principal results and optimism-corrected discrimination.
2	Background and rationale	Introduction, paragraphs 1–3.
3	Objectives	Introduction, final paragraph; descriptive and exploratory predictive objectives distinguished.
4	Study design	Methods 2.1; cross-sectional outcome assessment and definition of RRs as ratios of proportions.
5	Setting	Methods 2.2; dates and service context specified.
6	Participants	Methods 2.3; eligibility, exclusions and repeat procedures specified.
7	Variables	Methods 2.4–2.5; exposures, outcomes and covariates defined, with operational definitions from the study data dictionary and variables not available in the dataset listed.
8	Data sources and measurement	Methods 2.2, 2.4, 2.5 and 2.6.
9	Bias	Methods 2.7 and Discussion 4.7; causal structure in Supplementary Figure S1.
10	Study size	Methods 2.8; complete census used.
11	Quantitative variables	Methods 2.4 and 2.9; age groupings and continuous modelling explained.
12	Statistical methods	Methods 2.9; adjustment rationale, missing data (complete-case analysis with multiple-imputation sensitivity analysis), spline and ROC analyses with bootstrap internal validation and calibration. Item 12(e), sensitivity analyses of the robustness of the age estimate: Section 2.9, results in Section 3.6 and Table 6. The conditional sensitivity, specificity and predictive values of the age threshold (Sections 3.4–3.5) are diagnostic operating characteristics, not sensitivity analyses.
13	Participants	Results 3.1 and Figure 1; bounding analysis for the 37 excluded women in Section 3.1.
14	Descriptive data	Results 3.2 and Table 1; missingness described in Methods/Results, with age-specific denominators; complete and incomplete cases compared in Supplementary Table S6.
15	Outcome data	Results 3.3 and Table 2.
16	Main results	Results 3.4–3.6 and Tables 4 and 6; crude and adjusted estimates with 95% CIs in the same complete-case population; absolute differences with 95% CIs.
17	Other analyses	Age strata (Table 3), spline, EIN/carcinoma endpoint, ROC analyses with internal validation (Table 5) and sensitivity analyses (Table 6) in Results 3.4–3.6.
18	Key results	Discussion 4.1.
19	Limitations	Discussion 4.7.
20	Interpretation	Discussion 4.2–4.6.
21	Generalisability	Discussion 4.7–4.8.
22	Funding	Funding section.

Supplementary Table S2. RECORD extension reporting matrix

Item	RECORD domain	Location and implementation
1.1	Routinely collected data identified	Title/abstract and Methods 2.1–2.2 identify retrospective routine hospital data.
6.1	Population selection	Methods 2.3 provides explicit eligibility and exclusion rules.
6.2	Validation of selection	Methods 2.6 specifies independent verification of core eligibility fields.
7.1	Codes/algorithms	Clinical definitions and derived outcomes are specified in Methods 2.4–2.5; operational outcome coding is in Supplementary Table S4.
8.1	Data linkage	Methods 2.2 explains encounter-level linkage to pathology; no external linkage is used.
12.1	Access to study population	Procedure and pathology systems define the source population and denominator.
12.2	Data-cleaning methods	Methods 2.6 describes the predefined data dictionary, verification and reproducible derivation.
12.3	Linkage methods/evaluation	Not applicable beyond deterministic hospital encounter linkage; stated in Methods 2.2.
13.1	Selection flow	Figure 1 reports screened, excluded, eligible and analysed records.
14.1	Data availability	Data access is described in the Data Availability Statement.
19.1	Limitations of routine data	Discussion 4.7 addresses coding, documentation, selection and measurement limitations, including variables not available in the analytic dataset.
22.1	Access to protocol/code	Methods describes analytic choices; the data dictionary (codebook), coding rules (Supplementary Table S4) and the R code for the modified Poisson regression, sensitivity analyses and bootstrap procedures are available from the corresponding author on request. The de-identified patient-level dataset can be shared on reasonable request, subject to Ethics Committee approval and Regulation (EU) 2016/679 (Data Availability Statement). No separately registered protocol exists (Methods 2.1).

Supplementary Table S3. Selected guideline-related characteristics other than age, by age group

Characteristic	<45 years (n = 361)	≥45 years (n = 463)	p value
Persistent AUB (≥6 months)	171 (47.4%)	226 (48.8%)	0.725
Failed medical treatment (documented persistent bleeding after treatment)	167 (46.3%)	218 (47.1%)	0.833
Intermenstrual or mixed bleeding	128 (35.5%)	178 (38.4%)	0.384
Obesity (BMI ≥30 kg/m^2; recorded indicator when BMI missing, n = 17)	94 (26.0%)	155 (33.5%)	0.022
Chronic anovulation (including PCOS)	87 (24.1%)	107 (23.1%)	0.741
Obesity or chronic anovulation (including PCOS)	152 (42.1%)	218 (47.1%)	0.159
Suspected endometrial polyp	46 (12.7%)	97 (21.0%)	0.002
Tamoxifen exposure (current or previous)	0 (0.0%)	3 (0.6%)	0.261
Composite A: at least one of persistent AUB, failed medical treatment, obesity or chronic anovulation	291 (80.6%)	375 (81.0%)	0.929
Composite B: at least one of the characteristics listed above	318 (88.1%)	423 (91.4%)	0.130
Women <45 years without any Composite A characteristic (n = 70): broad outcome; EIN or carcinoma	4 (5.7%; 95% CI 2.2–13.8); 2 (2.9%; 0.8–9.8)	—	—
Women <45 years with at least one Composite A characteristic (n = 291): broad outcome; EIN or carcinoma	20 (6.9%; 95% CI 4.5–10.4); 2 (0.7%; 0.2–2.5)	—	—
Women <45 years without any listed characteristic (Composite B) (n = 43): broad outcome; EIN or carcinoma	3 (7.0%; 95% CI 2.4–18.6); 1 (2.3%; 0.4–12.1)	—	—

The specific indication for each procedure was not recorded as a structured variable; this table shows documented clinical characteristics that correspond to guideline indications other than age (ACOG and NICE; references 9–11 of the main manuscript). Characteristics are not mutually exclusive. Tamoxifen is reported separately because its endometrial effects are tissue-specific and differ from unopposed oestrogen exposure. Because only women who underwent sampling are included, similar frequencies in the two age groups do not show that the probability of being sampled was similar. 95% CIs are Wilson intervals. p values by Fisher's exact test. AUB, abnormal uterine bleeding; EIN, endometrial intraepithelial neoplasia; PCOS, polycystic ovary syndrome.

Supplementary Table S4. Operational coding of histopathology outcomes

Final histopathology	Primary outcome	EIN/carcinoma endpoint
Proliferative, secretory, disordered proliferative, atrophic/inactive, benign polyp, other benign	Benign	No
Hyperplasia without atypia	Significant pathology	No
EIN/atypical hyperplasia	Significant pathology	Yes
Endometrial carcinoma	Significant pathology	Yes

Coding notes. Each specimen was assigned a single final category from the routine report; no central review was performed. Hyperplasia was coded as without atypia or with atypia/EIN (two-tier WHO classification); disordered proliferative endometrium was coded as benign. When more than one finding was reported, the specimen was classified by the most severe lesion (carcinoma > EIN/atypical hyperplasia > hyperplasia without atypia > benign). The classification edition cited in each report, the wording of ambiguous reports and the presence of coexisting findings were not recorded in the analytic dataset.

Supplementary Table S5. Specification of the exploratory pre-biopsy logistic regression model (complete cases, n = 776; 76 events)

Term	Coding	β (SE)	OR (95% CI)
Intercept	—	-4.604 (0.524)	—
Age ≥45 years	1 = age ≥45 years; 0 = <45 years	0.538 (0.274)	1.71 (1.00–2.93)
Obesity	1 = BMI ≥30 kg/m^2	0.275 (0.262)	1.32 (0.79–2.20)
Diabetes mellitus	1 = documented pre-existing or type 2 diabetes	0.871 (0.441)	2.39 (1.01–5.68)
Chronic anovulation	1 = documented chronic anovulatory pattern (includes PCOS)	0.311 (0.278)	1.36 (0.79–2.35)
Nulliparity	1 = no previous birth	0.888 (0.266)	2.43 (1.44–4.09)
Intermenstrual or mixed bleeding	1 = intermenstrual or mixed bleeding	0.544 (0.253)	1.72 (1.05–2.83)
Endometrial thickness	Continuous, per 1 mm (linear)	0.136 (0.045)	1.15 (1.05–1.25)

Predicted probability = 1 / [1 + exp(−LP)], with LP = −4.604 + 0.538 × age ≥45 + 0.275 × obesity + 0.871 × diabetes + 0.311 × chronic anovulation + 0.888 × nulliparity + 0.544 × intermenstrual/mixed bleeding + 0.136 × endometrial thickness (mm). Apparent AUC 0.714 (95% CI 0.651–0.776); optimism-corrected AUC 0.686; optimism-corrected calibration-in-the-large 0.01 (approximate 95% interval −0.24 to 0.28) and calibration slope 0.87 (0.65–1.15) (1500 bootstrap resamples). Endometrial thickness entered linearly (a three-knot spline did not improve fit, p = 0.87). The model was developed only among women already selected for sampling, has not been externally validated and is not intended for clinical use. BMI, body mass index; OR, odds ratio; PCOS, polycystic ovary syndrome; SE, standard error.

Supplementary Table S6. Comparison of complete and incomplete cases among the 824 analysed women

Characteristic	Complete cases (n = 776)	Incomplete cases (n = 48)	p value
Age, years, mean (SD)	45.0 (5.9)	45.9 (5.4)	0.243
Age ≥45 years	434 (55.9%)	29 (60.4%)	0.653
Pipelle biopsy	488 (62.9%)	30 (62.5%)	1.000
Dilatation and curettage	188 (24.2%)	12 (25.0%)	
Hysteroscopic biopsy	100 (12.9%)	6 (12.5%)	
Broad outcome	76 (9.8%)	5 (10.4%)	0.804
EIN/atypical hyperplasia or carcinoma	27 (3.5%)	2 (4.2%)	0.684

Incomplete cases: BMI missing (n = 17) or endometrial thickness missing (n = 31); no woman was missing both. p values by Welch's t test (age) and Fisher's exact test (categorical variables; one test for sampling method). Patient-level data for the 37 women excluded because of inadequate or unavailable histopathology were not available in the analytic dataset and could not be included in this comparison. EIN, endometrial intraepithelial neoplasia; SD, standard deviation.

Supplementary Table S7. Histopathological findings and suspected polyps by sampling method

Finding	Pipelle biopsy (n = 518)	Dilatation and curettage (n = 200)	Hysteroscopic biopsy (n = 106)
Proliferative endometrium	145 (28.0%)	59 (29.5%)	30 (28.3%)
Secretory endometrium	98 (18.9%)	39 (19.5%)	14 (13.2%)

Disordered proliferative endometrium	95 (18.3%)	28 (14.0%)	27 (25.5%)
Benign endometrial polyp	61 (11.8%)	26 (13.0%)	14 (13.2%)
Atrophic or inactive endometrium	39 (7.5%)	18 (9.0%)	10 (9.4%)
Other benign histology	31 (6.0%)	6 (3.0%)	3 (2.8%)
Hyperplasia without atypia	33 (6.4%)	14 (7.0%)	5 (4.7%)
EIN or atypical hyperplasia	9 (1.7%)	6 (3.0%)	2 (1.9%)
Endometrial carcinoma	7 (1.4%)	4 (2.0%)	1 (0.9%)
Broad outcome (total)	49 (9.5%)	24 (12.0%)	8 (7.5%)
EIN or carcinoma (total)	16 (3.1%)	10 (5.0%)	3 (2.8%)
Polyp suspected before sampling	73 (14.1%)	38 (19.0%)	32 (30.2%)
Age ≥45 years	270 (52.1%)	121 (60.5%)	72 (67.9%)
Intermenstrual or mixed bleeding	187 (36.1%)	73 (36.5%)	46 (43.4%)

Percentages are calculated among women with adequate histopathology in each sampling group. The frequency of inadequate specimens by procedure could not be reported because data on the excluded women were not available in the analytic dataset. EIN, endometrial intraepithelial neoplasia.

Supplementary Figures

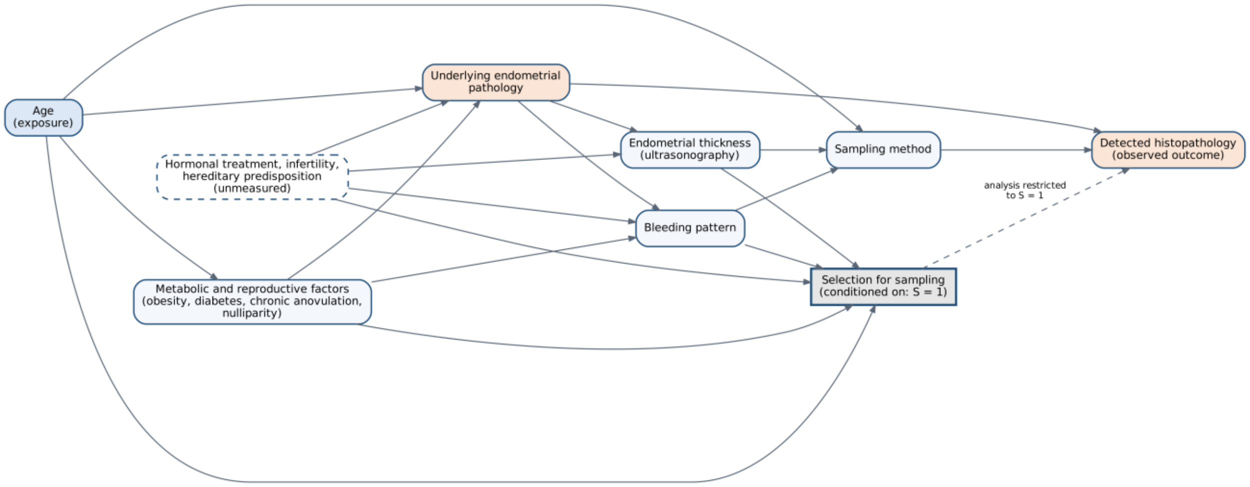

Figure S1. Assumed causal structure (directed acyclic graph). Age may influence endometrial pathology directly and through metabolic and reproductive factors. Bleeding pattern and endometrial thickness are partly consequences of the pathology and, together with age, influence selection for sampling and the choice of sampling method. Because only sampled women (S = 1) are analysed, selection acts as a collider between age and factors related to pathology, which can bias the age association in a way that multivariable adjustment cannot remove. Adjusting for bleeding pattern, endometrial thickness or sampling method may also absorb part of the age association; models omitting these variables are reported in Table 6. Unmeasured factors are shown with a dashed border.

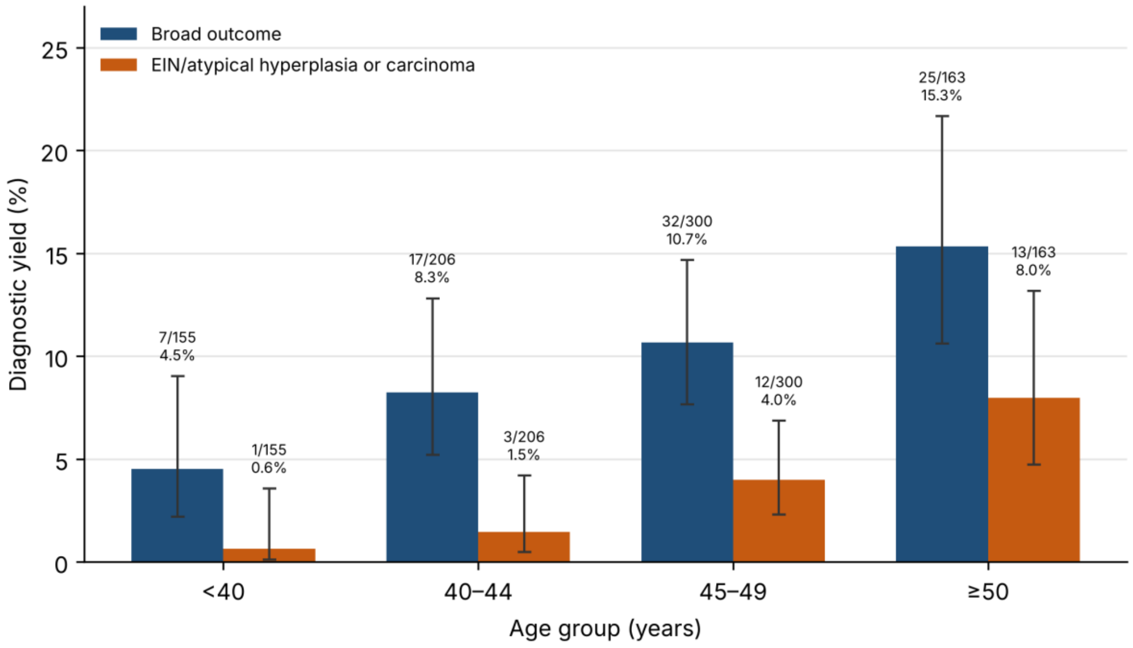

Figure S2. Age-stratified diagnostic yield of the broad outcome (hyperplasia without atypia, EIN/atypical hyperplasia or carcinoma) and of EIN/atypical hyperplasia or carcinoma among women who underwent sampling (n = 824). Error bars are Wilson 95% confidence intervals.

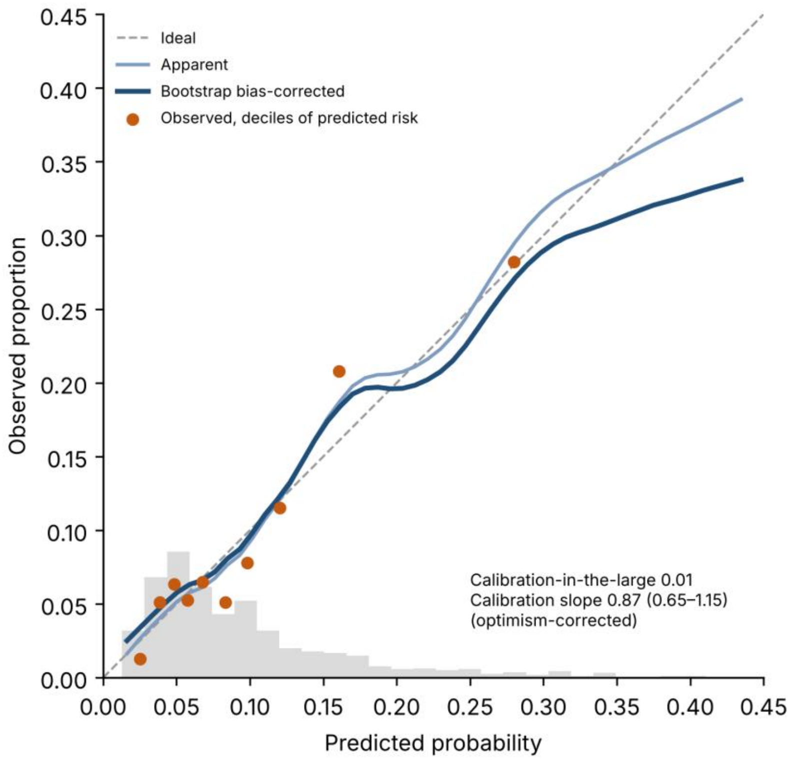

Figure S3. Calibration of the exploratory pre-biopsy model in the 776 complete cases. The dashed diagonal represents ideal calibration; the light line shows the apparent and the dark line the bootstrap bias-corrected (1500 resamples) smoothed relation between predicted and observed probabilities; points show observed proportions by deciles of predicted risk; the grey histogram shows the distribution of predicted probabilities. Optimism-corrected calibration-in-the-large 0.01 (approximate 95% interval −0.24 to 0.28); calibration slope 0.87 (0.65–1.15), indicating overfitting. The deviation at predicted probabilities above about 0.20 occurs where data are sparse. Obesity is defined as BMI ≥30 kg/m^2.
